# PSME2 offers value as a biomarker of M1 macrophage infiltration in pan-cancer and inhibits osteosarcoma malignant phenotypes

**DOI:** 10.7150/ijbs.90226

**Published:** 2024-02-04

**Authors:** Ruoqi Li, Lei Yan, Jingwei Jiu, Haifeng Liu, Dijun Li, Xiaoke Li, Jing Zhang, Songyan Li, Zijuan Fan, Zhi Lv, Yuanyuan Zhu, Bin Wang

**Affiliations:** 1 Department of Orthopaedic Surgery, The First Affliated Hospital, Zhejiang University School of Medicine, Hangzhou, China.; 2 General Surgery Department, Third Hospital of Shanxi Medical University, Shanxi Bethune Hospital, Shanxi Academy of Medical Sciences, Tongji Shanxi Hospital, Taiyuan, Shanxi, China.; 3 Department of orthopedics, The Second Hospital of Shanxi Medical University, Shanxi Key laboratory of Bone and Soft Tissue injury repair, 382 Wuyi Road, Taiyuan, Shanxi, China.; 4 Department of Emergency, The Affiliated Hospital of Guizhou Medical University, Guiyang, Guizhou 550001, China.; 5 Clinical College of Medicine, Guizhou Medical University, Guiyang, Guizhou 550025, China.; 6 Department of Health Statistics, School of Public Health, Shanxi Medical University, Shanxi, China.; 7 State Key Laboratory for Diagnosis and Treatment of Infectious Diseases, Collaborative Innovation Center for Diagnosis and Treatment of Infectious Diseases, The First Affiliated Hospital, Zhejiang University School of Medicine, Hangzhou, Zhejiang, China.

**Keywords:** Pan-cancer analysis, M1 macrophage, genome instability, immunotherapy, Molecular Docking

## Abstract

A growing number of studies have revealed an association between proteasome activator complex subunit 2 (PSME2) and the progression of various forms of cancer. However, the effect of PSME2 on osteosarcoma progression is unknown. Pan-cancer analyses focused on the immunological activity and prognostic relevance of PSME2 have yet to be conducted. The Cancer Genome Atlas and Genome-Tissue Expression databases were leveraged to evaluate PSME2 expression and activity across 33 cancer types. Significant PSME2 dysregulation was noted in a wide range of cancer types and this gene was found to offer significant diagnostic and prognostic utility in most analyzed cancers. From a mechanistic perspective, PSME2 expression levels were correlated with DNA methylation, DNA repair, genomic instability, and TME scores in multiple cancer types. PSME2 was subsequently established as a pan-cancer biomarker of M1 macrophage infiltration based on a combination of bulk, single-cell, and spatial transcriptomic data and confirmatory fluorescent staining results. In osteosarcoma cells, overexpressing PSME2 significantly suppressed tumor proliferative, migratory, and invasive activity. Screening efforts also successfully identified the PSME2-activating drug irinotecan, which can synergistically promote the death of osteosarcoma cells when combined with the chemotherapeutic drug paclitaxel. As a biomarker of M1 macrophage infiltration, PSME2 expression levels may offer insight into tumor development and progression for a wide range of cancers including osteosarcoma, emphasizing its potential utility as a prognostic and therapeutic target worthy of further study.

## Introduction

As a key regulator of 20S proteasomal activity, proteasome activator 28 (PA28) plays vital roles in the control of transcriptional activity, antigen presentation, cell cycle progression, learning, memory, and the suppression of depressive behaviors 1-3. PA28 is composed of three protein subunits known as PA28α, PA28β (also known as PSME2), and PA28γ. In healthy individuals, PSME2 is reportedly inactive 4. However, recent evidence suggests that PSME2 plays a valuable role in regulating tumor development and the presentation of tumor-derived antigens. When overexpressed, PSME2 is capable of suppressing the tumorigenic activity of both the TE-1 esophageal squamous cell carcinoma cell line as well as the MKN45 gastric adenocarcinoma (GA) cell line, consistent with its potential relevance as a therapeutic target in a range of cancers 5-7. Functionally, PSME2 is capable of promoting the presentation of tumor-derived antigens on MHC class I molecules 8, thereby facilitating, for example, the presentation of the TRP2 360-368 epitope in melanoma to enable the activation of cytotoxic T lymphocytes (CTLs) reactive to this antigenic moiety 9.

While the above data provide support for a potential model in which PSME2 may influence a range of cancer types, systematic pan-cancer research evaluating the roles played by PSME2 is currently lacking 5-7. Research focused on only a single cancer type has the potential to overlook the mechanistic importance of a given target gene owing to the lack of a corresponding global perspective. There is thus a pressing need to examine the important roles that the expression of PSME2 plays in many different forms of cancer in order to inform future experimental and clinical research efforts.

**Figure 1:**
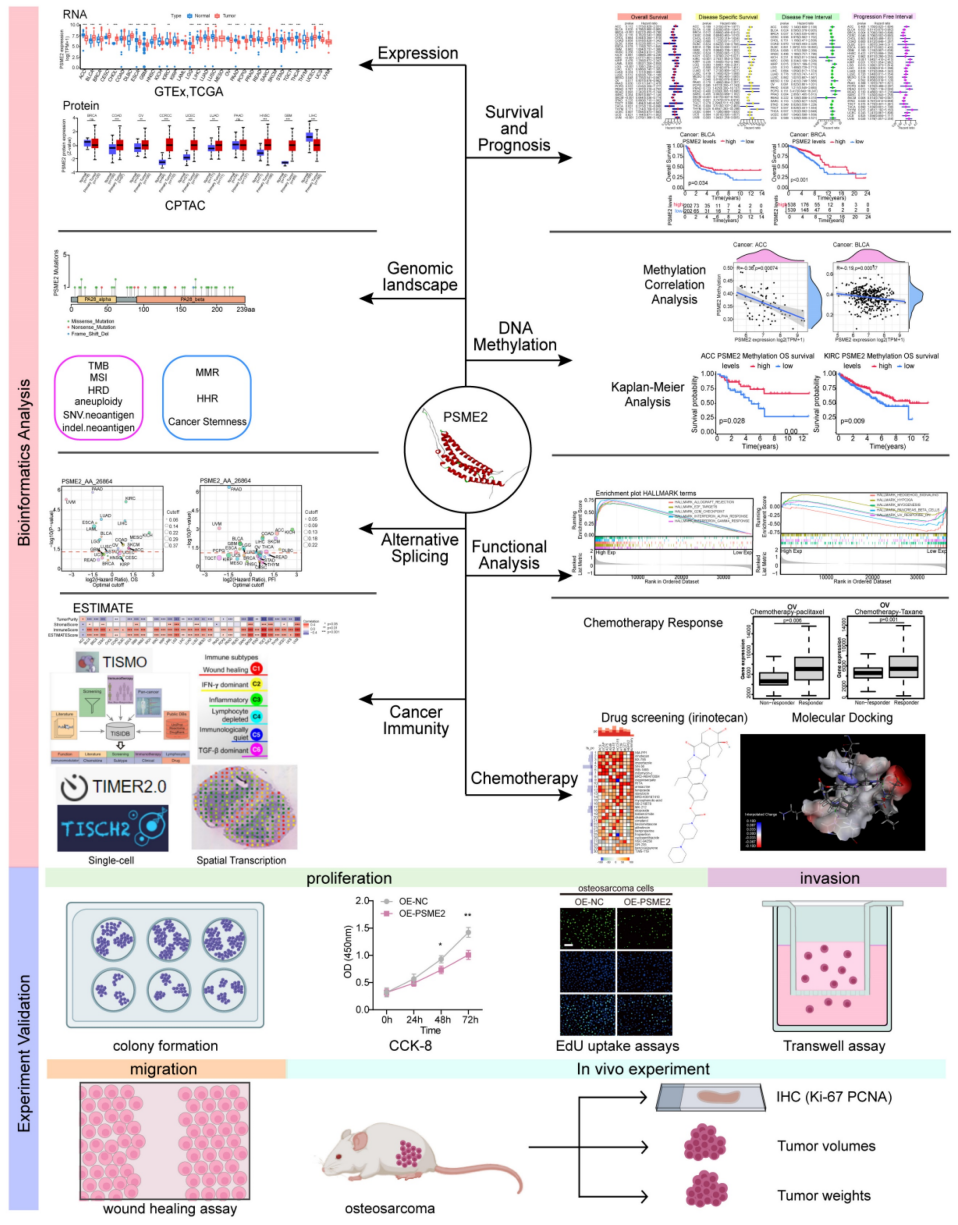
Study flowchart.

Here, a poly-omics pan-cancer study of PSME2 was conducted using an integrated series of tools and datasets corresponding to different cancers and normal tissues in an effort to clarify the relationships between this gene, clinical characteristics, and multi-omic heterogeneity. In particular, these analyses centered around DNA repair, DNA damage, and the induction of cancer-related immune responses, with subsequent confirmation of the identification of PSME2 as a biomarker of M1 macrophage infiltration through fluorescent staining. The effects of PSME2 on the malignant phenotype of osteosarcoma were verified by* in vivo* and *in vitro* experiments. Screening efforts were also used to identify PSME2-activating drugs with potential value for use in specific forms of cancer. An overview of the experimental approaches employed herein is provided in Figure 1.

## Materials and Methods

### Pan-Cancer Data Collection and Processing

The UCSC Xena platform was used for accessing The Cancer Genome Atlas (TCGA) and Genotype-Tissue Expression (GTEx) databases pertaining to pan-cancer PSME2 expression levels and corresponding clinical characteristics 10. A dataset comprising long-read transcriptome sequencing of osteosarcoma (GSE218035) was obtained from the Gene Expression Omnibus. PSME2 expression in different cancer cell lines was analyzed using the Cancer Cell Line Encyclopedia (CCLE) database 11. The cBioPortal for Cancer Genomics database was used as a source of single-nucleotide variation (SNV) data across cancer types and for merged HM27 and HM450 methylation data 12. A log2 (transcripts per million (TPM)+1) transformation approach was used for the normalization of transcriptional data. For details regarding the naming and abbreviations of the 33 tumor types included in this study, see Supplementary Table 1. PSME2 protein FASTA sequences were obtained from the NCBI protein database.

### Pan-Cancer Analyses of Differential PSME2 Expression

Data from the TCGA and GTEx databases were merged to compare PSME2 mRNA levels between tumors and normal tissues for 33 cancer types. The R 'ggplot2' package was used for analyses of differential gene expression. Boxplots were used to present differences in expression levels across cancer subtypes or stages. PSME2 protein levels were compared between tumors and normal tissues with Clinical Proteomic Tumor Analysis Consortium data derived from the UALCAN portal 13.

### Diagnostic and Prognostic Analysis

The R 'pROC' package was used to generate receiver operating characteristic (ROC) curves for cancers of interest, and survival outcomes were compared between individuals expressing low and high levels of PSME2 with Kaplan-Meier curves, stratifying patients according to median PSME2 expression. The R 'survival' and 'survminer' packages were used for survival curve generation. Univariate Cox regression analyses performed in the R 'survival' and 'forestplot' packages were then used to evaluate the prognostic relevance of PSME2 expression as a predictor of overall survival (OS), disease-specific survival (DSS), progression-free interval (PFI), and disease-free interval (DFI).

### Genomic Alteration and Mutational Burden Analyses

Pan-cancer analyses of the frequencies of genomic mutations, amplifications, and deep deletions were conducted with the cBioPortal Cancer Type Summary module 14. Processed SNV data were evaluated with reference to PSME2 protein domains identified with the R 'maftools' package to highlight the mutational landscape of this gene in pan-cancer datasets 15. The Tumor Immune Dysfunction and Exclusion (TIDE) 16 Copy_Number module was used to obtain Kaplan-Meier curves for use in evaluating the prognostic relevance of PSME2 copy number variations (CNVs). The R 'maftools' package was used to assess tumor mutational burden (TMB), while aneuploidy, neoantigen, homologous recombination deficiency (HRD), and microsatellite instability (MSI)-related data were obtained from prior reports 17, with correlations between these characteristics and PSME2 expression then being assessed.

### DNA Mismatch Repair, Stemness, and Epigenetic Modification Analyses

Visual analyses of the relationship between the expression of PSME2, five mismatch repair (MMR) genes 18, and four DNA methyltransferases (DNMTs) 19 were conducted. In addition, the ARIEL3 clinical trial 20 was used to retrieve 30 homologous recombination repair (HRR)-related genes that were evaluated for correlations with PSME2 mRNA levels using the GEPIA2 tool 21. Differentially methylated probes-based stemness index (DMPsi) values for individual cancer types were derived from a prior report 22, and the associations between these values and PSME2 expression at the mRNA level were also assessed. Associations between PSME2 and the expression of 44 N1‐methyladenosine (m1A), 5‐methylcytosine (m5C), and N6‐methyladenosine (m6A) modifying genes were evaluated with heat maps 23.

### PSME2 DNA Methylation Analyses

Using methylation data derived from cBioPortal, correlations between the methylation of PSME2 and patient OS, DSS, DFI, and PFI were assessed with the R 'survival' package. The R 'ggpubr' package was used to facilitate the visual examination of the link between PSME2 promote methylation and the expression of this gene at the mRNA level. The TIDE Methylation module was used to assess the relationship between the methylation of the PSME2 promoter and CTLs.

### PSME2 Alternative Splicing Analyses

Clinically relevant PSME2 alternative splicing (AS) was examined with the OncoSplicing server 24 using the ClinicalAS tool, searching for relevant PSME2 AS events in the SpliceSeq and SplAdder projects. The percent spliced-in (PSI) data for TCGA cancer and GTEx tissue data were displayed with PanPlot, and PSI differences were compared between tumors and corresponding normal tissues for AS events present in >3 cancer types.

### Functional Enrichment and Interaction Analyses

The STRING 25 database was used to construct a protein-protein interaction network for PSME2 based. UALCAN was used to explore pan-cancer pathway-level somatic alterations for key pathways, while GEPIA2 was used to evaluate correlative relationships between PSME2 expression levels and pathway-related signatures 26. The GEPIA2 Similar Gene Detection function was used to select the top 100 genes co-expressed with PSME2, and these genes were used for Gene Ontology (GO) enrichment analyses using the R 'clusterProfiler' package with the use of the R 'org.Hs.eg.db' package to obtain GO annotations 27. A false discovery rate corrected P < 0.05 served as the significance threshold. Gene set enrichment analysis (GSEA) results were also used to quantitatively assess PSME2 functional enrichment 28. Tumor samples were separated into groups exhibiting low and high levels of PSME2 expression based on median expression levels, and a hallmark pathway GSEA approach was implemented by downloading the h.all.v7.4.symbols.gmt gene set from the Molecular Signatures Database 29.

### Pan-Cancer Analyses of the Immunological Roles of PSME2

The ESTIMATE algorithm was used to compute Immune, Stromal, and ESTIMATE score values for 33 cancer types 30. Correlations between PSME2 and previously identified immune checkpoint markers were also examined at the mRNA level 31. PSME2 expression was also evaluated in tumors classified into 6 immunological subtypes (C1: Wound healing; C2: IFN-γ dominant; C3: Inflammatory; C4: Lymphocyte-depleted; C5: Immunologically quiet; C6: TGF-β dominant) using the TISIDB subtype module 32. Correlative relationships between PSME2 and immune-associated genes (MHCs, chemokine receptors, chemokines, immunosuppressive genes, and immunostimulatory genes) were examined in a range of cancer types. The effects of cytokine treatment on PSME2 expression levels were assessed with the online Tumor Immune Syngeneic MOuse tool (TISMO) 33, enabling the evaluating of samples exposed to cytokine, anti-CLTA4, or anti-PD-L1 treatment.

The xCell, MCPCOUNTER, and CIBERSORT algorithms were used to quantify the relative proportions of infiltrating immune cells 34-36. In addition, data on immune cell infiltration derived from single-sample gene set enrichment analysis were obtained from the ImmuCellAI database 37. Spearman's correlation coefficients were used to explore the association between PSME2 expression and the relative abundance of different types of infiltrating immune cells. The TIMER2.0 database was also used to explore the correlation between PSME2 expression and macrophage infiltration 38. Spatially resolved transcriptomic data available through the SpatialDB tool 39 were employed to examine spatial relationships pertaining to the expression of PSME2, the general macrophage marker CD68, and the M1 macrophage marker TLR2 in breast cancer (BC) and melanoma. Single-cell-resolution analyses of PSME2 in various cancers were performed with the Tumor Immune Single-cell Hub (TISCH) 15. Correlations between PSME2 and 14 functional states in cancers were assessed with single-cell sequencing data from the CancerSEA 'correlation plot' module 40.

### PSME2-activating Drug Screening and Molecular Docking analyses

The potential relevance of PSME2 to the treatment of BC, glioblastoma (GBM), and ovarian cancer (OV) was evaluated by comparing the expression of this gene between non-responders and responders when assessing therapy-associated survival outcomes with ROC plotter 41. Potential PSME2-activating compounds were screened with the cMap 'query' tool 42, using the top 100 most highly upregulated and downregulated genes identified when comparing PSME2-high and PSME2-low groups (stratified according to median PSME2 expression). The top 30 compounds were then plotted with a heatmap, and their mechanisms of action (MoA) were evaluated. The expression of PSME2 and concentrations associated with 50% growth inhibition (GI50) in various cell lines were then plotted for top compounds with the NCI Developmental Therapeutics Program COMPARE tool. PSME2 protein homology modeling was conducted with the AlphaFold2 software 43, with SAVES v6.0 being used for rank_1 protein estimation and molecular docking analyses that were subsequently conducted with Discovery Studio v19.1.0. Following the automated preparation of PSME2 and candidate compounds of interest, binding sites and compound conformations were assessed, and LibDock was used for docking. Those sites exhibiting the highest LibDockScore values and molecular conformations were then selected for additional analyses of 3D binding pocket interactions and 2D intermolecular force distances.

### Experimental Methods

The experimental methods are shown in Supplementary Material 2.

### Statistical Analysis

R v4.2.1 was used for all statistical analyses. Results were compared between groups with one-way ANOVAs or Student's t-tests. Kaplan-Meier curves and log-rank tests or Cox proportional hazard regression models were employed when conducting survival analyses. Pearson or Spearman correlation coefficient values were used to evaluate relationships between variables, with |r| = 0.3 being considered indicative of a relevant correlative relationship. Significant correlations between PSME2 and DNMT expression were considered present if correlations were significant for any 2 of the 4 analyzed DNMTs and none of the other DNMTs exhibited opposing statistical results. P < 0.05 was the cut-off threshold when defining significance (*P < 0.05, **P < 0.01, ***P < 0.001; ns: not significant).

## Results

### Pan-Cancer Analyses of PSME2 Expression

**Figure 2:**
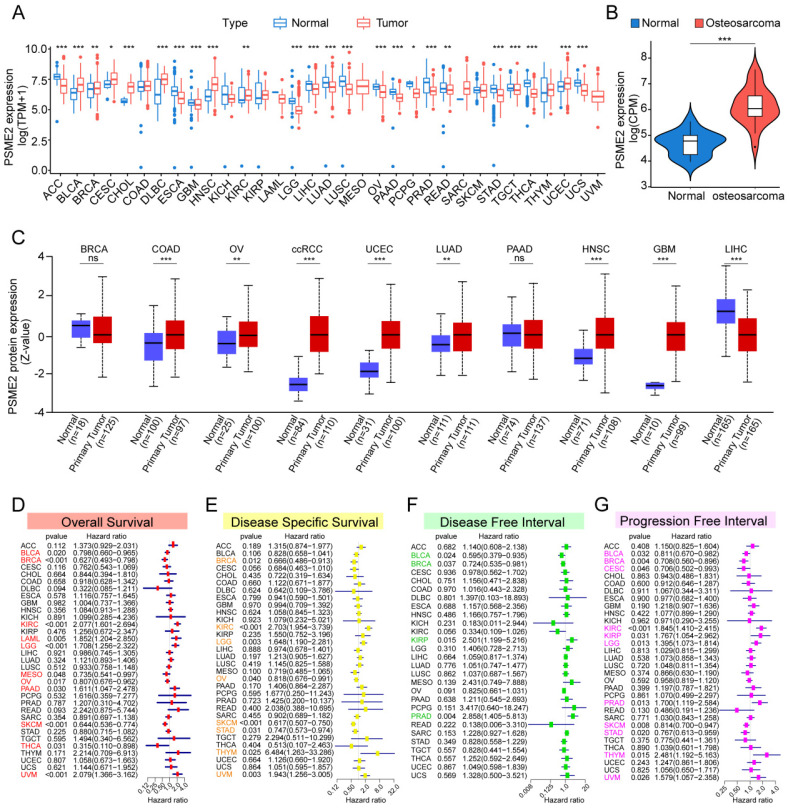
** Pan-cancer analyses of PSME2 expression and prognostic relevance.** (A) The TCGA and GTEx datasets were used for integrated analyses of PSME2 expression in tumors and healthy tissues. (B) PSME2 mRNA expression in osteosarcoma compared with the adjacent normal tissues in the GSE218035 dataset. (C) UALCAN analyses of PSME2 protein levels in primary tumors and normal tissues. (D-G) Forest plots were used for pan-cancer analyses of PSME2 and OS (D), DSS (E), DFI (F), and PFI (G). *P < 0.05, **P < 0.01, ***P < 0.001; ns: not significant. Abbreviations: CPM, counts per million.

The TCGA and GTEx databases were initially used to conduct a systematic pan-cancer analysis of the expression of PSME2 at the mRNA level. This approach revealed differential PSME2 expression in 24 cancer types (Figure 2A, Figure S1A). Osteosarcoma tissues exhibited higher PSME2 expression compared to adjacent normal tissues (Figure 2B). The relative PSME2 expression levels across different cell lines, obtained from CCLE data, are presented in Figure S1B. Data from the UALCAN database revealed protein-level PSME2 upregulation in COAD, OV, clear cell renal cell carcinoma, UCEC, LUAD, HNSC, and GBM, whereas the downregulation of this protein was evident in LIHC (Figure 2C). PSME2 expression was also evaluated in 33 tumor types across a range of clinical stages, subtypes, and TNM stages (Figure S2).

### PSME2 Offers Diagnostic and Prognostic Utility in Different Cancers

ROC curves suggested that PSME2 may offer utility as a diagnostic biomarker in certain cancers (Figure S3). The prognostic relevance of PSME2 as a predictor of prognostic outcomes including OSS, DSS, PFI, and DFI was then assessed for 33 tumor types in the TCGA database. Univariate Cox regression analyses indicated that PSME2 was significantly associated with poorer OS in KIRC, LAML, LGG, PAAD, and UVM, whereas it was a protective factor in BLCA, BRCA, MESO, OV, SKCM, and THCA (Figure 2D). PSME2 was also a risk factor significantly associated with worse DSS in KIRC, LGG, and THYM, while it was a protective factor in BRCA, OV, SKCM, STAD (Figure 2E). With respect to DFI, PSME2 was a risk factor in KIRP and PRAD whereas it was protective in BLCA and BRCA (Figure 2F). With respect to PFI, PSME2 was a risk factor in KIRC, KIRP, LGG, PRAD, THYM, and UVM, while it was protective in BLCA, BRCA, CESC, SKCM, and STAD (Figure 2G). Kaplan-Meier curves were also used to evaluate these four prognostic outcomes (Figure S4). Overall these results suggested that lower levels of PSME2 expression were generally related to worse prognostic outcomes in BLCA, BRCA, OV, SKCM, and THCA patients.

### Analyses of PSME2 Genomic Alterations and Genomic Instability

**Figure 3:**
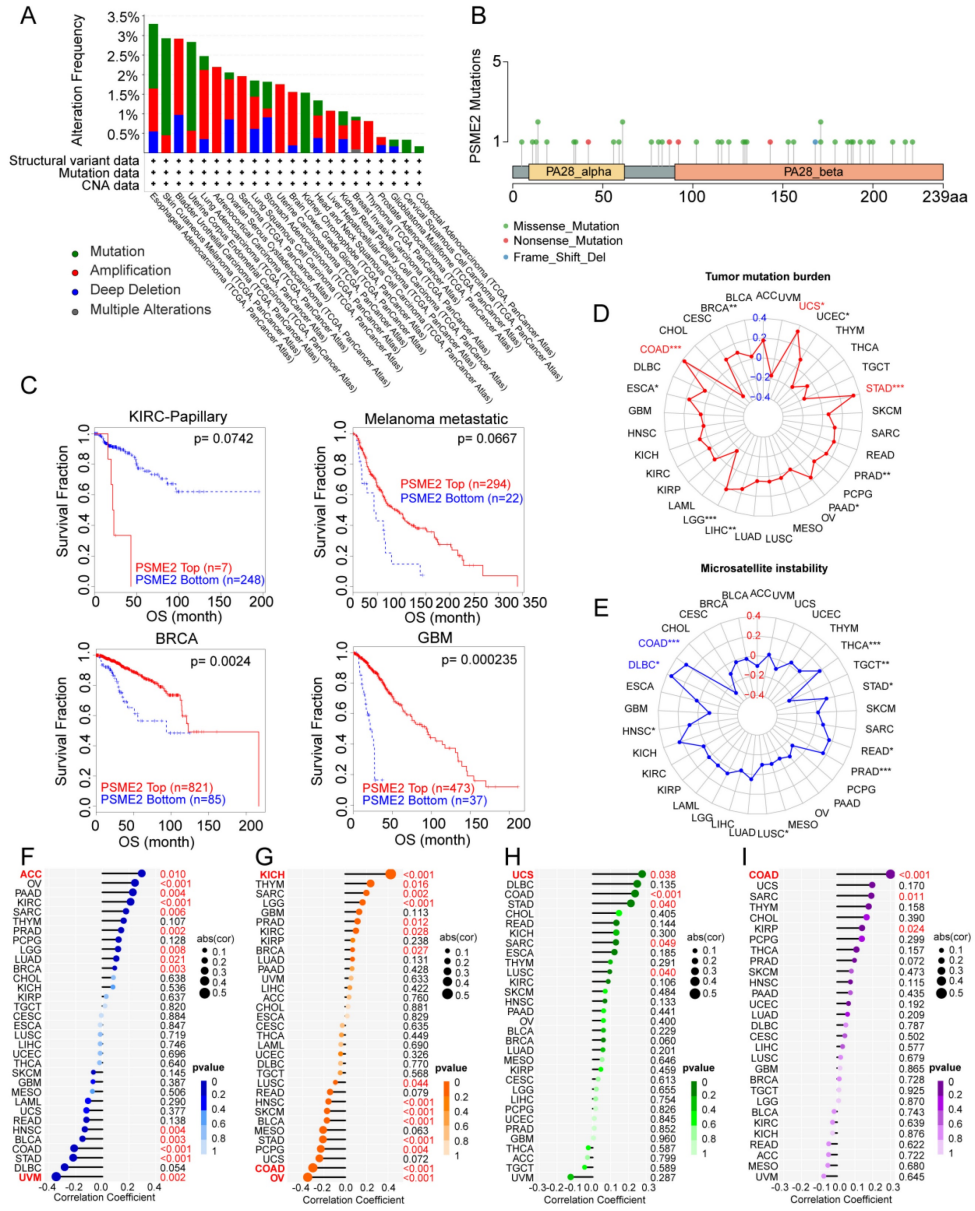
** PSME2 expression is correlated with genomic instability.** (A) Pan-cancer analyses of genomic changes in PSME2 in the TCGA database were conducted, including analyses of mutations, amplifications, and deep deletions. (B) The pan-cancer PSME2 SNV landscape, including missense, frameshift deletion, and splice site mutations. (C) The prognostic relevance of PSME2 CNVs and survival in four cancers was assessed using Kaplan-Meier curves generated with the TIDE tool. (D, E) Radar charts representing pan-cancer analyses of the link between PSME2 and both TMB (D) and MSI (E). The red font indicates a correlation coefficient of ≥0.3 and p-value < 0.05 between PSME2 expression and TMB. Blue font indicates a correlation coefficient of ≥0.3 and p-value <0.05 between PSME2 expression and MSI. (F-I) Lollipop charts were used to visualize correlations between PSME2 levels and HRD (F), aneuploidy (G), SNV.neoantigens (H), and Indel.neoantigens (I), with dot sizes being proportional to sample sizes and dot color being proportional to p-values. Cancers with p-values <0.05 and |a correlation coefficient| ≥ 0.3 are shown in red bold type, with regular red font indicating that the cancer meets the p-value < 0.05 threshold.

Genomics strategies provide a powerful means of analyzing cancer 44. To assess potential genome-level alterations in PSME2 in specific cancers, a pan-cancer analysis of PSME2 CNVs and SNVs was conducted. PSME2 amplification was primarily detected in BLCA, LUAD, ACC, SARC, UCS, LGG, LIHC, BRCA, and THCA, whereas deep deletions were common in STAD, and high rates of SNVs were evident in SKCM, UCEC, and KICH (Figures 3A, B). When TIDE analyses were conducted based on levels of these CNVs, patients with metastatic melanoma, GBM, and BRCA exhibiting high PSME2 CNV levels exhibited higher survival rates, whereas the opposite was true in papillary KIRC (Figure 3C). Correlations between PSME2 and TMB, MSI, HRD, aneuploidy, and neoantigens were also evaluated given the abundance of such mutations in tumors and their potential effects on prognostic and therapeutic outcomes 45, 46. A positive correlation was detected between PSME2 and TMB in COAD, STAD, and UCS (Figure 3D), and between PSME2 and MSI in COAD and DLBC (Figure 3E). PSME2 was negatively and positively correlated with HRD in UVM and ACC, respectively (Figure 3F). Similarly, a positive correlation was detected between PSME2 and aneuploidy in KICH whereas a negative correlative relationship was detected in OV and COAD (Figure 3G). Tumor-specific neoantigens represent important targets for the induction of an antitumor immune response such that they are associated with prognostic outcomes for a range of cancers 47. SNVs and insertion/deletion (indel) mutations were used to identify putative MHC-binding neoantigens capable of inducing an adaptive antitumor immune response 17. The only correlation between PSME2 and neoantigens was with indel neoantigens in COAD (Figure 3H-3I). Overall these data suggest an important association between PSME2 and genomic instability.

### PSME2 Levels are Associated with DNA Repair, Methylation, and Cancer Cell Stem-like Characteristics

**Figure 4:**
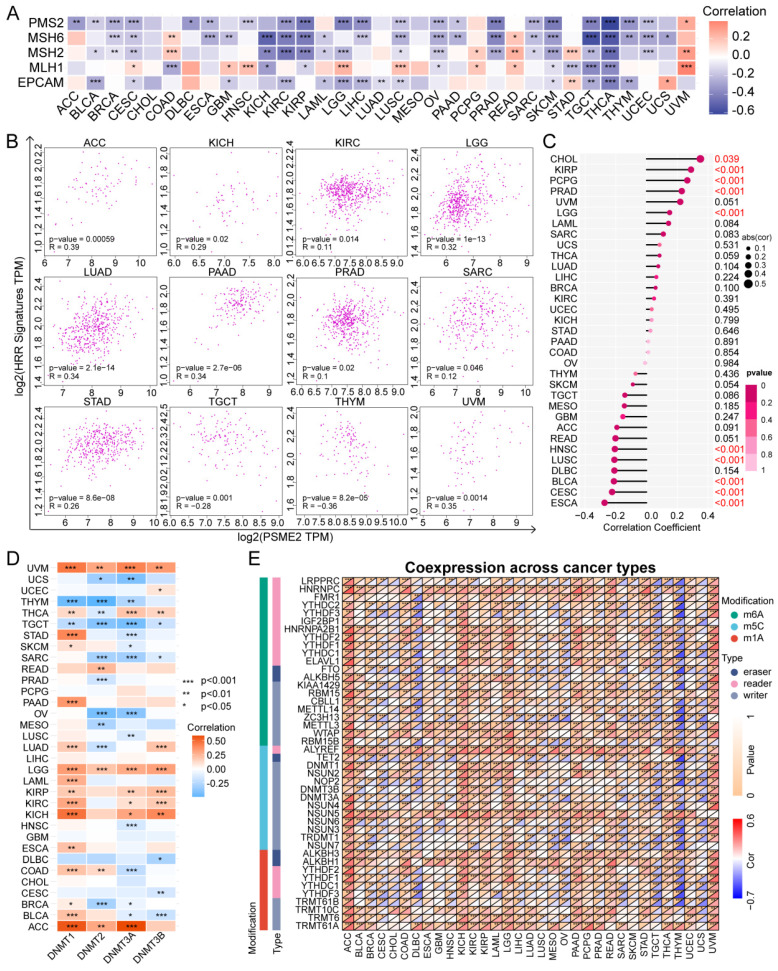
** PSME2 is associated with DNA repair, epigenetic modifications, and stemness.** (A) Associations between PSME2 and five MMR genes across cancer types are presented in a heatmap. (B) Correlation scatter plots for 12 cancers highlighting correlations between a 30-gene HRR signature and the levels of PSME2. (C) Correlations between PSME2 levels and stemness are presented with a lollipop chart in which dot sizes are proportional to sample size and colors are indicative of p-values. (D) Correlations between PSME2 and four DNMTS are presented in a heatmap. (E) Correlations between pan-cancer RNA modulations and PSME2 levels are presented in a heatmap. *P < 0.05, **P < 0.01, ***P < 0.001.

The DNA damage response is a complex series of mechanisms responsible for maintaining the stability and integrity of the genome by detecting and eliminating abnormal sequences and structures within chromosomes 48. Tumor cells develop mechanisms that enable them to evade certain therapeutic strategies by co-opting the MMR 49 and HRR 50 mechanisms, endowing these tumor cells with stem-like self-maintenance abilities 51. As such, the associations between PSME2 expression levels and MMR-associated genes, HRR signatures, and stemness were next evaluated. A negative correlation was detected between PSME2 and the expression of a range of MMR genes in cancers including BLCA, BRCA, KICH, KIRC, KIRP, LIHC, OV, PRAD, SARC, SKCM, TGCT, THYM, and UCEC, with a particularly pronounced relationship in THCA (Figure 4A). A positive correlation was also evident between PSME2 and HRR signatures in ACC, LGG, LUAD, PAAD, and UVM, whereas this correlation was negative in THYM (Figure 4B). PSME2 was additionally positively correlated with tumor stemness in CHOL (Figure 4C). These results thus suggest a role for PSME2 as a regulator of cancer progression through its ability to influence DNA damage repair.

Epigenetic modifications also play an important role in shaping the onset and progression of cancers such that they are increasingly popular targets for researchers 52. DNMTs are responsible for catalyzing DNA methylation, potentially modulating the proliferation, differentiation, survival, and cell cycle progression characteristics of tumor cells 53. PSME2 expression was significantly negatively correlated with DNMT levels in UCS, THYM, TGCT, SARC, and OV, whereas the opposite was true in UVM, LGG, KIRP, KIRC, KICH, and ACC (Figure 4D). Notably, we observed a negative correlation between PSME2 mRNA expression and methylation (Figure S5), and survival outcomes assessed by Kaplan-Meier curves showed that reduced methylation was predictive of shorter survival in ACC and PRAD (Figure S6A). Relationships between PSME2 promoter methylation and CTLs were also assessed with TIDE in DLBC, STAD, OV, CESC, LUAD, and triple-negative breast cancer (TNBC) (Figure S6B). Correlative relationships between the expression of PSME2 and RNA modulator genes were also examined (Figure 4E). Together, these analyses suggested a role for PSME2 in DNA methylation and mRNA modification in a range of cancer types.

### Alternative PSME2 Splicing is Associated with Survival Outcomes

**Figure 5:**
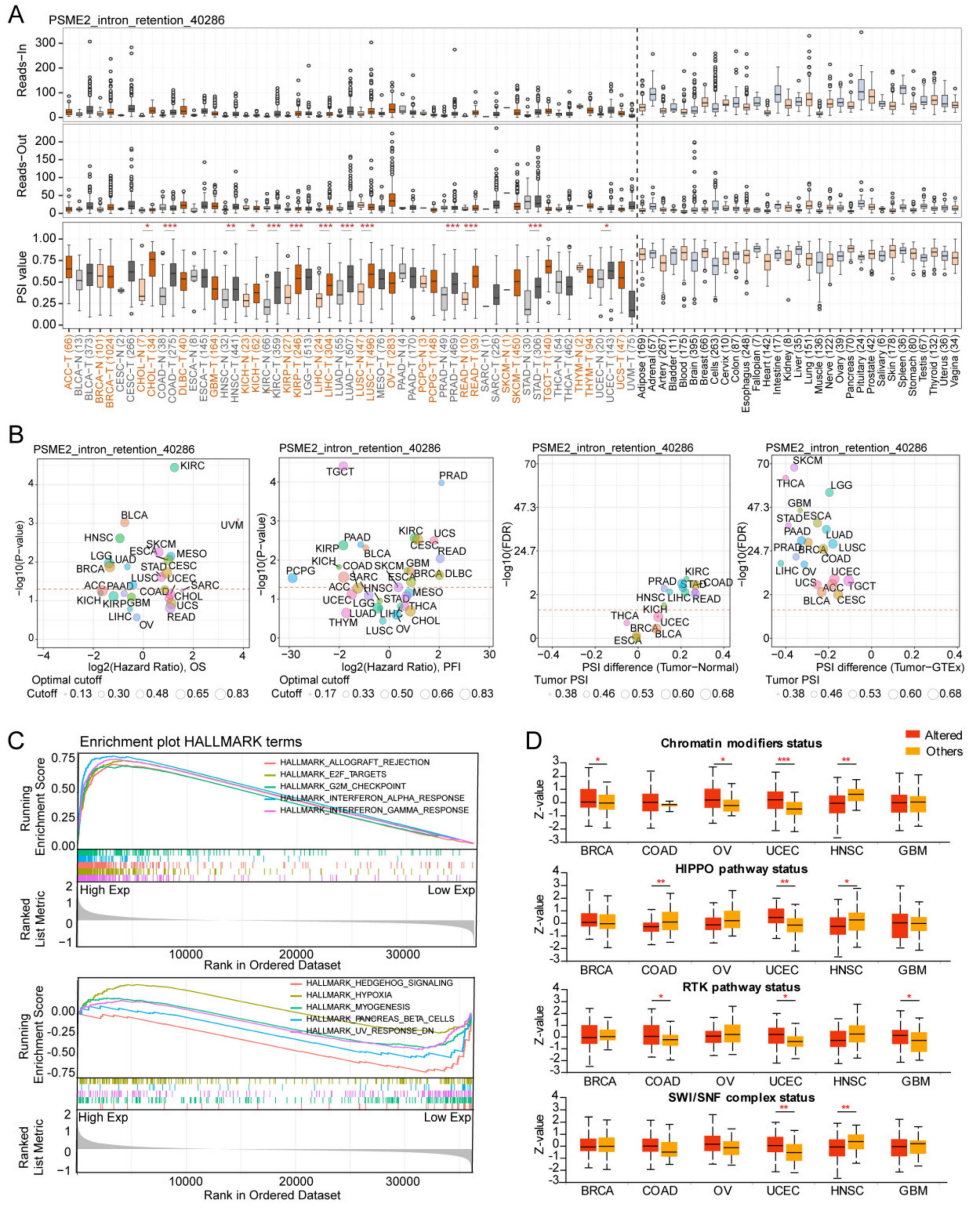
** Alternative PSME2 splicing events and associations with immune and oncogenic pathways.** (A) Reads-in, reads-out, and PSI values PSME2_ .Intron_Retention_40286 in pan-cancers, adjacent samples, and healthy tissue samples. Colored labels correspond to tumors and matching adjacent tissues, while non-tumor tissues are labeled in black. (B) PSI differences when comparing tumors and corresponding healthy or adjacent tissues and the association between PSME2_ Intron_Retention_40286 events and prognosis. *P < 0.05, **P < 0.01, ***P < 0.001. (C) Pan-cancer HALLMARK GSEA enrichment plots. For these analyses, samples were stratified according to median PSME2 expression. (D) Box plots comparing the expression of PSME2 in 6 cancers based on the presence or absence of somatic alterations in the indicated pathways as assessed with the UALCAN tool.

Specific changes in gene splicing can occur in tumor cells that can support disease progression, and the identification of these AS events has the potential to support the prognostic and diagnostic evaluation of patients 54. Using OncoSplicing, 51 clinically relevant PSME2 AS events were detected (Table S2), including PSME2_intron retention_40286 in the TCGA SpIAdderSeq database and the PSME2_AA_26864 event in the TCGA SpliceSeq database. Pan-cancer analysis results showing the PSI of PSME2_intron retention_40286 event are provided in Figure 5A. Relative to normal samples, a higher PSI was evident in CHOL, HNSC, KIRC, LIHC, LUAD, and STAD, whereas the opposite was true in BRCA, KICH, THYM, and UCEC. Figure 5B summarizes the statistical results of PSI differences between tumor and normal/adjacent tissues and the relationship between them and the prognosis. For further details regarding the PSME2_AA_26864 event, see Figure S7A-S7B. Together these results suggest that PSME2 AS events may have important implications for the progression of many cancers.

### PSME2 is Linked to Immune Activity and is Involved in Multiple Oncogenic Pathways

To gain insight into the functions of PSME2 in tumor cells, a functional enrichment analysis was employed. Using the STRING tool, 10 proteins with experimentally validated interactions with PSME2 were identified (Figure S7C). GEPIA2 data were then used to identify the top 100 genes co-expressed with PSME2, and GO enrichment analyses of these genes highlighted a close association between PSME2 and various immune-related activities (Figure S7D, Table S3).

HALLMARK GSEA results further confirmed a close link between PSME2 and both immunological activity and cell cycle progression (Figure 5C, Table S4). It was subsequently observed that PSME2 expression was increased in patients with UCEC showing somatic alterations in chromatin modifications, HIPPO and RTK pathways, and SWI/SNF complex status, while PSME2 expression was decreased in HNSC patients with somatic alterations in chromatin modifications, the HIPPO pathway, or SWI/SNF complex status (Figure 5D). The correlations between PSME2 expression and these pathway-related signatures were also explored 26, revealing a consistent correlation between PSME2 and these signatures (Figure S8, Table S5). The results indicated the potential involvement of PSME2 in multiple oncogenic pathways in a variety of cancers.

### PSME2 is Associated with Immune Cell Infiltration and Cytokine-Related Activity in Tumors

**Figure 6:**
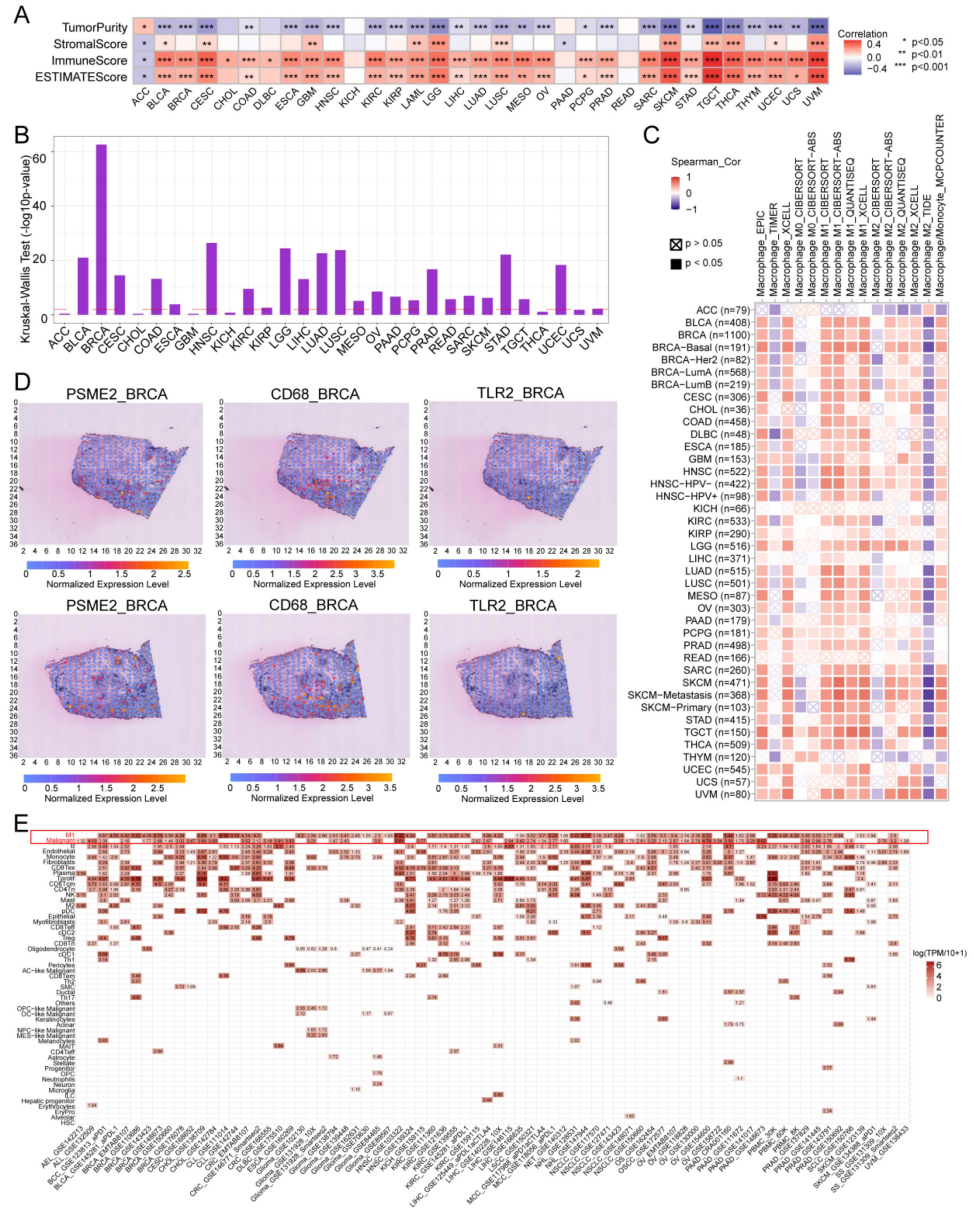
** PSME2 is a pan-cancer biomarker of M1 macrophage infiltration.** (A) Correlations between PSME2 levels and ESTIMATE, Immune, and Stromal scores were presented using a heatmap. (B) Correlations between PSME2 and immune subtypes were assessed with the TSIDB tool. The red dashed line represents p-value = 0.05. (C) TIMER2.0 determined the correlations between PSME2 expression and macrophage infiltration levels in pan-cancer using multiple algorithms. (D) Spatial transcriptomic sections were analyzed to assess the overlapping patterns of PSME2, CD68, and TLR2 expression. Dots are colored based on the expression levels for the indicated genes. (E) The TISCH tool was used to assess PSME2 expression levels in cancer-derived single-cell clusters.

The ESTIMATE algorithm was next implemented to evaluate the association between PSME2 and immunological characteristics for 33 tumor types. A positive correlation between ESTIMATE and Immune score values was evident for most cancer types, although the opposite was true for ACC (Figure 6A), with Figure S9A presenting the top 6 cancers exhibiting the strongest correlations. Differential PSME2 expression in various immunological cancer subtypes was further assessed with TISIDB, revealing a significant relationship between PSME2 levels and immune subtypes for 23 cancer types (Figure 5B, Figure S9B). Specifically, PSME2 upregulation was evident in BRCA, LUAD, HNSC, LUSC, and STAD tumors of the C2 subtype, suggesting a functional relationship between this gene and IFN-γ signaling activity. A pan-cancer analysis of associations between PSME2 and immune-related genes was also conducted (Figure S10), and TISMO was used to compare the expression of PSME2 between cancer cell lines treated *in vitro* with cytokines (Figure S11A) and in samples before and after *in vivo* anti-PD-1 and anti-CTLA4 treatment (Figure S11B). Higher PSME2 expression levels were evident in samples in the responder group following cytokine, anti-PD1, and anti-CTLA4 treatment.

### PSME2 is a Biomarker of Infiltration by M1 Macrophages

To better understand the association between PSME2 expression and cancer-related immune activity, correlations between PSME2 expression and the infiltration of various immune cell types were assessed (Figure S12, Figure S13A). TIMER2.0 analyses revealed a positive correlation between M1 macrophage infiltration and PSME2 expression levels across cancers (Figure 6C), while a low correlation was found with PSME2 promoter methylation (Figure S13B, Table S6). Spatial transcriptomic data from Spatial DB further confirmed that PSME2 expression patterns overlapped substantially with those of the macrophage marker CD68 and the M1 macrophage marker TLR2 in BRCA and melanoma (Figure 6D, Figure S14A), implied potential co-localization of these genes. Single-cell transcriptional data from TISCH additionally confirmed the expression of PSME2 in M1 macrophages and malignant tumor cells in most analyzed cancer types (Figure 6E).

**Figure 7:**
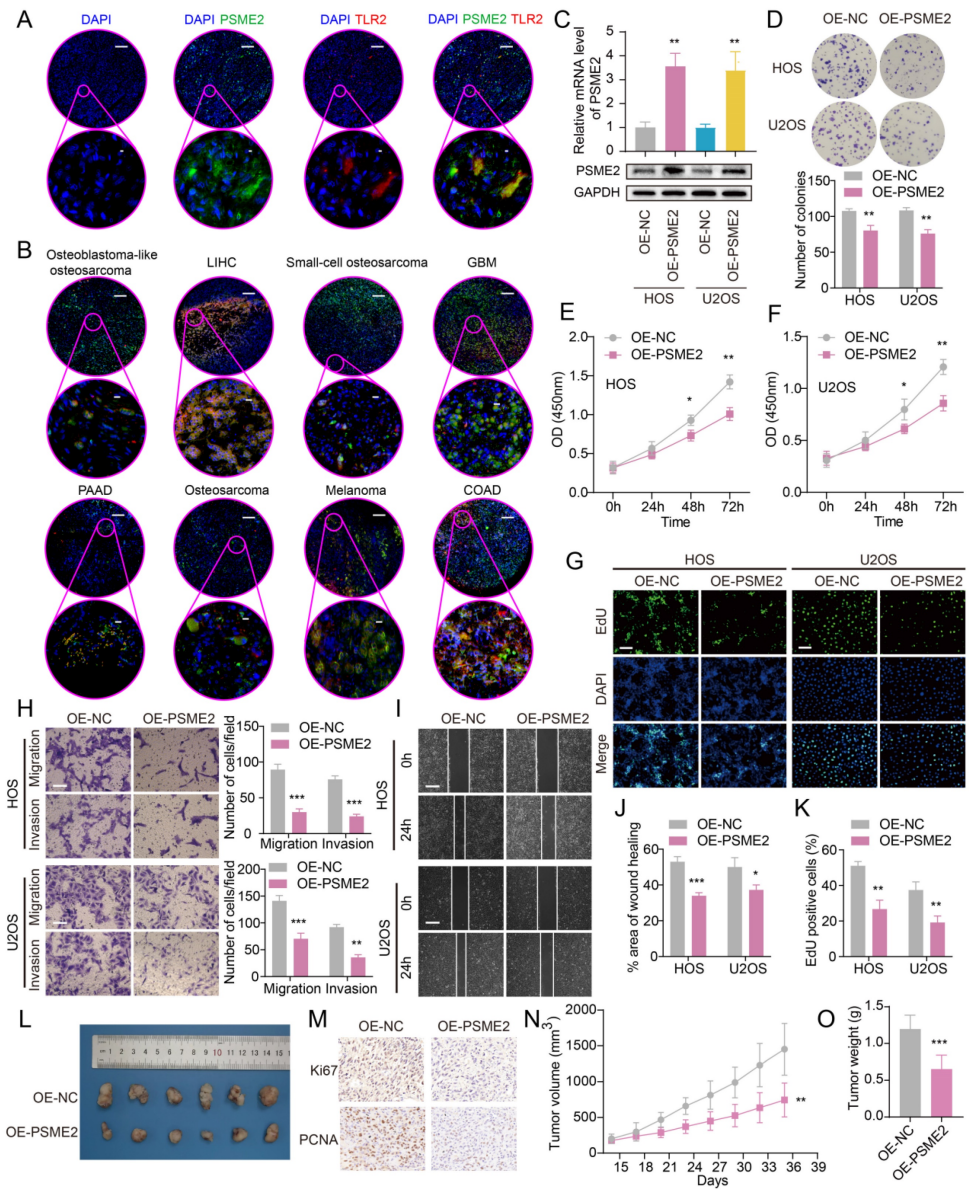
** Evaluation of the ability of PSME2 to regulate OS tumor cell malignancy.** (A) Images of tumor sections stained for TLR2 (red) or PSME2 (green), with DAPI (blue) counterstaining. The pink areas in the upper images (Scale bar: 200 μm) are magnified below (Scale bar: 5 μm). (B) Merged staining results for samples presented as in (A) (Scale bar for magnified images: 10 μm). (C) mRNA and protein levels of PSME2 in transfected cells. (D-G, K) The impact of PSME2 on the proliferation of tumor cells was assessed through colony formation, CCK-8, and EdU uptake assays (Scale bar: 400 μm). (H) Tumor cell migration and invasion were assessed with a Transwell assay approach. (I, J) Tumor cell migration following PSME2 overexpression was assessed through a wound healing assay approach. (L) Tumor images. (M) Tumor immunohistochemical staining for PCNA and Ki-67. (N) Tumor volumes. (O) Tumor weights. *P < 0.05, **P < 0.01, ***P < 0.001.

To experimentally verify these results, fluorescent staining was conducted for these different marker proteins in a range of paraffin-embedded cancer sections. Clear PSME2 and TLR2 co-expression was evident in COAD, GBM, LIHC, PAAD, osteosarcoma, and melanoma (Figure 7A-7B). The bulk, spatial, single-cell transcriptional data and the fluorescence staining results above highlight the close relationship between PSME2 expression and M1 macrophages, suggesting that PSME2 may be a pan-cancer biomarker of M1 macrophage infiltration.

CancerSEA single-cell sequencing data were also used to assess correlative relationships between PSME2 expression and 14 cancer functional states, revealing positive associations between PSME2 and the cell cycle, DNA repair, and DNA damage in non-small cell lung cancer (Figure S14B, Table S7), highlighting these as possible processes through which PSME2 shapes oncogenic progression.

### PSME2 Suppresses Osteosarcoma Cell Growth and Malignancy

Next, the ability of PSME2 to influence the malignant characteristics of osteosarcoma cells was assessed to expand upon the above findings using the U2OS and HOS cell lines. Both qPCR and Western blotting were used to confirm successful PSME2 overexpression in these cells (Figure 7C). This overexpression markedly suppressed the proliferative activity of both of these cell lines (Figure 7D-G, K), while also inhibiting their ability to engage in migratory and invasive behaviors in wound healing and Transwell assays (Figure 7H-J). *In vivo,* PSME2 overexpression also suppressed subcutaneous xenograft tumor growth relative to control tumors (Figure 7L-O). Together, these results support the ability of PSME2 to significantly suppress osteosarcoma tumor proliferative, migratory, and invasive activity.

### The PSME2-Activating Drug Irinotecan Acts Synergistically with Paclitaxel to Inhibit Osteosarcoma Proliferation

**Figure 8:**
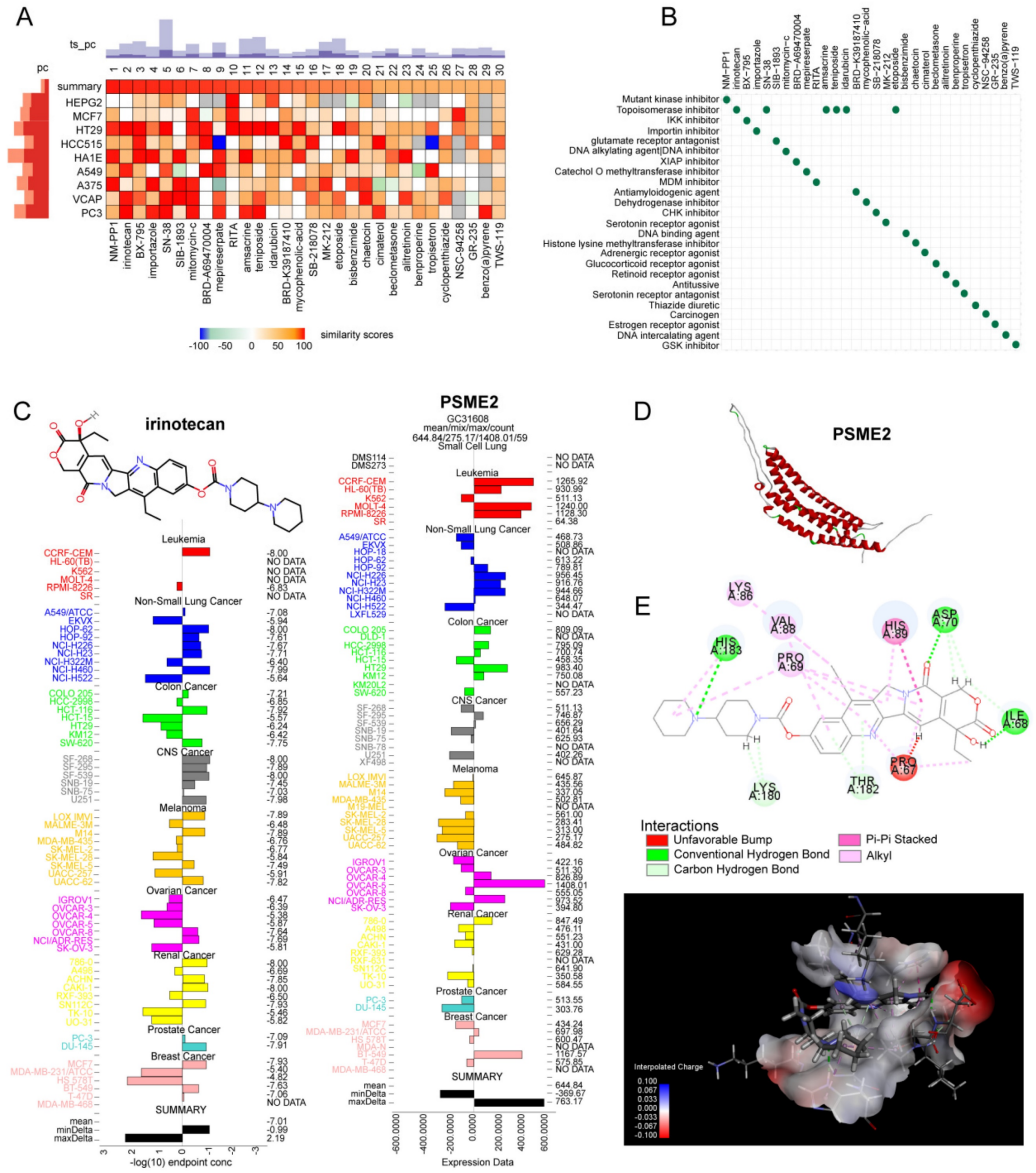
** Evaluation of the association between PSME2 and therapeutic outcomes, PSME2-activating drug identification, and molecular docking analyses.** (A) A heatmap compiling the top 30 compounds associated with consistent transcriptional changes to those impacted by median PSME2 expression grouping. Similarity scores are represented with colors. pc denotes the percent of total perturbagens, querying the column sample against all rows, that exceed the given thresholds. The height of the light orange bar indicates % connections ≥95 and the height of the dark orange bar indicates % connections ≥97.5. ts_pc denotes the percent of total Touchstone perturbagens that connect to the selected row perturbagen above the indicated thresholds. The height of the light blue bar represents % connections ≥95 and the height of the dark blue bar represents % connections ≥97.5. (B) MoA scatter plots representing the MoAs for the top 30 compounds shown in (A). (C) The NCI60 project was used to assess irinotecan GI50 values (left) and PSME2 levels in cell lines (right). The central line corresponds to mean -log10(GI50) or PSME2 expression values. (D) alphaFold2.0 was used to construct a model of PSME2. (E) The top 3D PSME2 structure constructed by homology modeling, with the upper left images showing the PSME2 pocket for drug binding. Candidate drug 2D structural characteristics, interacting amino acid residues, molecular forces, and molecular distances are also presented. Abbreviations: HIS, histidine; LYS, lysine; VAL, valine; PRO, proline; ASP, aspartic acid; THR, threonine; ILE, isoleucine.

The identification of medications that activate PSME2 is essential. The cMap tool was used to filter candidate compounds, revealing 30 potential PSME2-activating compounds based on consistent changes in transcriptional expression thereof in 9 cell lines (Figure 8A). Of these compounds, 6 were topoisomerase inhibitors, suggesting a close link between PSME2 and specific mechanisms within tumor cells (Figure 8B). With the COMPARE tool, the GI50 values for these compounds when treating a range of cancer cell lines were assessed, excluding NM-PPI due to a lack of testing data. For irinotecan, the average -log10(GI50) was -7.01, and high levels of PSME2 expression in BC cells were associated with a lower GI50 (Figure 8C). To assess the potential for binding between the PSME2 protein and irinotecan, a molecular docking study was conducted. Five models of PSME2 were constructed with alphaFold2.0 based on the obtained FASTA sequence (Supplementary Material 1), with the top-ranked model exhibiting an Overall Quality Factor of 91.85 (Figure 8D). Discovery Studio v19.1.0 was then used to perform molecular docking analyses, revealing the successful docking of irinotecan with PSME2, with a maximum LibDockScore of 125.562 (Figure 8E). This suggests that irinotecan is a candidate for the activation of PSME2.

**Figure 9:**
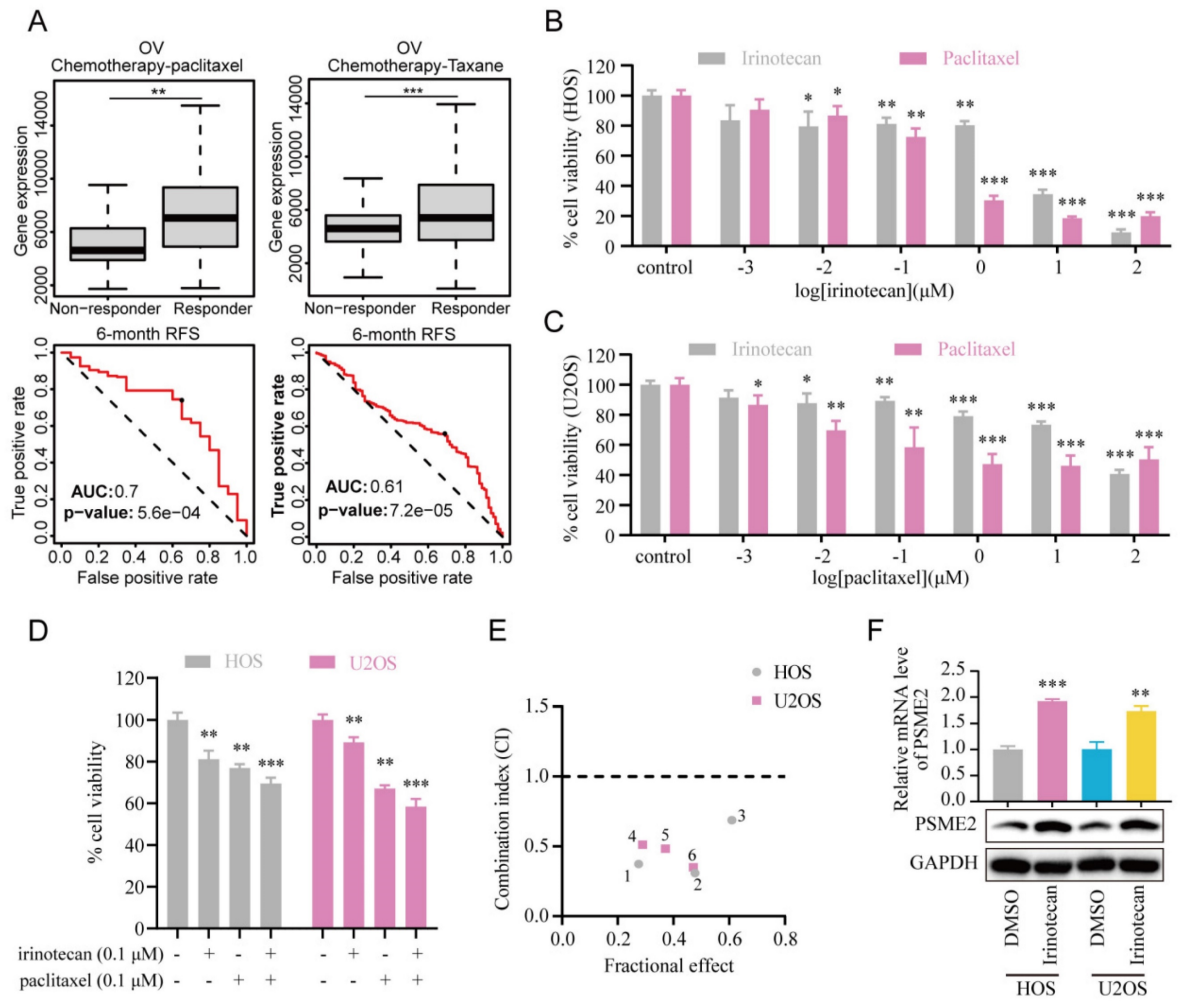
** PSME2 activator irinotecan acts synergistically with paclitaxel in inhibiting the proliferation of osteosarcoma cells.** (A) Box plots showing differences in PSME2 expressiont between responders and non-responders, with ROC curves corresponding to the ability of PSME2 levels to predict patient therapeutic responses, as assessed with the online ROCplotter tool. (B) Dose-dependent cytotoxicity of irinotecan and paclitaxel in HOS cells. (C) Dose-dependent cytotoxicity of irinotecan and paclitaxel in U2OS cells. (D) Cytotoxic effect of the combination of 0.1The cμM irinotecan and 0.1 μM paclitaxel in HOS and U2OS cells. (E) Osteosarcoma cells were co-treated with different concentrations of irinotecan and paclitaxel for 48 h. Combination index (CI) was determined by the Chou-Talalay method. (F) Alterations in PSME2 mRNA and protein levels after treatment of osteosarcoma cells with 10 µM irinotecan.

Subsequently, the association between PSME2 expression levels and chemotherapy response in cancer was assessed using ROCplotter. This showed that PSME2 was highly expressed in patients with OV that responded to chemotherapeutic drugs, including paclitaxel and taxane, with respective areas under the curve for 5-year RFS of 0.7 and 0.61 (Figure 9A). As OV patients with low levels of PSME2 expression responded poorly to routine chemotherapy, the possibility that the PSME2-activating medication irinotecan may increase tumor chemosensitivity was investigated. Paclitaxel is a successful chemotherapeutic drug commonly used to treat a variety of cancers. However, although the drug shows high initial efficacy, repeated usage invariably leads to resistance 55. Then, we investigated the effects of irinotecan combined with chemotherapeutic drugs on osteosarcoma cells, using paclitaxel as an example. It was found that both irinotecan and paclitaxel substantially and dose-dependently suppressed osteosarcoma cell growth (Figure 9B-9C). It was also found that subcytotoxic combinations of paclitaxel and irinotecan resulted in cell death, indicating synergistic interactions between the drugs (Figure 9D-9E, Table S8). Interestingly, irinotecan confirmed this hypothesis by activating PSME2 expression at both the mRNA and protein levels (Figure 9F). These findings provide support for the use of irinotecan as a PSME2 activator in combination with chemotherapeutic agents to improve the treatment of cancers.

## Discussion

Many prior studies have extensively characterized PSME2, revealing that it plays important roles in shaping transcriptional regulation, cell cycle progression, 20S proteasome activity, learning, memory, immune response induction, and the suppression of depressive behaviors 56. Recently, this protein has also been suggested to serve as a specific regulator of tumor-related behaviors for oncogenic progression in cancers such as ESCA 6, GA 5, and BRCA 8. Building on these prior reports, the present study was conducted to systematically analyze the expression, prognostic relevance, and functions of PSME2 across cancer types.

PSME2 was down-regulated at the mRNA level and up-regulated at the protein level in OV, LUAD, LIHC, and GBM tumor tissues compared to corresponding normal tissues. The phenomenon may be related to post-transcriptional and post-translational modifications of PSME2. In hepatocellular carcinoma, Snail mRNA was destabilized and degraded by METTL3 (m6A methyltransferase), with METTL3 binding to methylated Snail mRNA via YTHDF1 and eEF-2 and triggering the translational elongation of Snail mRNA 57. Similarly, in METTL3-deficient acute myeloid leukemia cells, c-MYC, Bcl-2, and PTEN protein levels were reduced despite a 2-5 log2-fold increase in mRNA expression 58. In the present study, PSME2 was found to be associated with the expression of a variety of genes associated with RNA modification, suggesting that the discrepancy between the mRNA and protein expression of PSME2 may be the result of post-transcriptional modifications. In addition, the regulation of TEM8 protein bythe E3 ubiquitin ligase ASB10 in triple-negative breast cancer tissues resulted in higher levels of TEM8 protein than in other subtypes of breast cancer, while the transcript levels were lower than in luminal breast cancer, suggesting that post-translational modification is also responsible for this discrepancy between the protein and mRNA expression levels 59. However, extensive experimental verification is needed to determine whether PSME2 is regulated by post-translational modifications. This provides a direction for subsequent investigations into the mechanisms underlying the role of PSME2 in cancer.

Moreover, PSME2 appears to function as a protective factor in the prognosis of SKCM, BRCA, and BLCA, while acting as a prognostic risk factor in KIRC, LGG, and UVM. This dual role of PSME2 may be attributed to the activation of distinct molecular pathways in different cancer types, underscoring the inherent complexity and heterogeneity characteristic of cancer biology. PSME2 was also identified as a potential pan-cancer biomarker for M1 macrophage infiltration. Lastly, screening and molecular docking studies were used to identify candidate drugs for PSME2 activation. It is noteworthy that the largest proportion of these candidate drugs were found to be topoisomerase inhibitors. Topoisomerases are enzymes that cleave one or both of the sugar-phosphate backbones of double-stranded DNA without altering its chemical composition 60. The activities of topoisomerases are known to be markedly increased in rapidly dividing cancer cells 61. Numerous critical cellular processes, such as transcription, chromosomal condensation, recombination, and DNA replication, depend on topoisomerases 61. Topoisomerase inhibitors can cause DNA damage and death in cancer cells by capturing covalent complexes of topoisomerase, suggesting that PSME2 may inhibit cancer through the process of DNA damage.

Immune checkpoint inhibitor (ICI) drugs including antibodies specific for CTLA-4, PD-1, and PD-L1 are the most effective and routinely used form of immunotherapy in clinical settings. TMB and MSI are parameters that can be used to predict tumor sensitivity to these drugs, with TMB- or MSI-high tumors being more likely to respond to ICIs 62. Here, a positive association was noted between PSME2 expression and TMB, MSI, and immune checkpoint expression in most analyzed cancer types. Following the administration of ICIs (anti-PD1, anti-PDL1, anti-CTLA4), responders tended to exhibit higher PSME2 levels relative to non-responders. This suggests that higher PSME2 levels in most cancers coincide with higher TMB and MSI levels, contributing to improved therapeutic outcomes following the administration of immunotherapeutic agents. Impaired PSME2 expression has previously been tied to the ability of colon tumors to avoid immunosurveillance 63. In fibroblasts overexpressing the proto-oncogene HER-2/neu, the impairment of PSME2 expression or function also impacted the efficacy of HER-2/neu-targeted T-cell-based immunotherapy, contributing to drug resistance 8. PSME2 may thus represent an important predictor of tumor immune status.

In GO enrichment analyses, PSME2 was related to innate immune response regulation and cytokine-mediated signaling. When evaluating six different therapeutically relevant tumor types, high levels of PSME2 expression tended to be evident in cancers of the IFN-γ-dominated C2 subtype. As a major inflammatory cytokine, IFNγ plays a key role in shaping immune response induction, tumor immunosurveillance, and the maintenance of normal tissue homeostasis 64. IFNγ signals through a JAK/STAT1 pathway to promote the upregulation of a range of interferon-stimulated genes with important immune effector activities 64. GSEA results in the present study also supported the potential ability of PSME2 to influence tumor development or progression by regulating IFN-α and IFN-γ responses. Such a mechanism for the PSME2-mediated regulation of tumor development has not previously been reported, highlighting a promising avenue for future study.

M1 macrophages are associated with more robust antitumor properties in contrast to pro-tumorigenic M2 macrophages such that therapies capable of promoting M2-to-M1 macrophage polarization may contribute to improved patient outcomes. Indeed, there is *in vivo* experimental evidence that inducing the M1 polarization of M2 macrophages can suppress angiogenesis and BC tumor growth 65. Here, higher levels of PSME2 expression were positively correlated with M1 macrophage infiltration in both bulk and single-cell transcriptomic datasets, and immunofluorescent staining confirmed the co-expression of PSME2 and M1 macrophage marker proteins. This strongly supports a role for PSME2 as a regulator of antitumor immunity in many types of cancer. As such, potential PSME2-activating drugs were identified for potential use in combination with existing therapies with the goal of enhancing tumor chemosensitivity.

## Supplementary Material

Supplementary figures and tables.

## Abbreviations

PSME2: proteasome activator complex subunit 2
PA28: proteasome activator 28
GA: gastric adenocarcinoma
CTLs: cytotoxic T lymphocytes
TCGA: The Cancer Genome Atlas
GTEx: Genotype-Tissue Expression
CCLE: the Cancer Cell Line Encyclopedia
SNV: single-nucleotide variation
TPM: transcripts per million
ROC: receiver operating characteristic
OS: overall survival
DSS: disease-specific survival
PFI: progression-free interval
DFI: disease-free interval
TIDE: Tumor Immune Dysfunction and Exclusion
CNV: copy number variation
TMB: tumor mutational burden
HRD: homologous recombination deficiency
MSI: microsatellite instability
MMR: mismatch repair
DNMTs: DNA methyltransferases
HRR: homologous recombination repair
DMPsi: Differentially methylated probes-based stemness index
m1A: N1‐methyladenosine
m5C: 5‐methylcytosine
m6A: N6‐methyladenosine
AS: alternative splicing
PSI: the percent spliced-in
GO: Gene Ontology
GSEA: Gene set enrichment analysis
TISMO: Tumor Immune Syngeneic MOuse tool
BC: breast cancer
TISCH: Tumor Immune Single-cell Hub
GBM: glioblastoma
OV: ovarian cancer
MoA: mechanisms of action
GI50: 50% growth inhibition
BLCA: bladder urothelial carcinoma
BRCA: breast invasive carcinoma
CESC: cervical squamous cell carcinoma and endocervical adenocarcinoma
CHOL: cholangiocarcinoma
DLBC: lymphoid neoplasm diffuse large B-cell lymphoma
HNSC: head and neck squamous cell carcinoma
KIRC: kidney renal clear cell carcinoma
UCEC: uterine corpus endometrial carcinoma
ACC: adrenocortical carcinoma
ESCA: esophageal carcinoma
LGG: brain lower-grade glioma
LIHC: liver hepatocellular carcinoma
LUAD: lung adenocarcinoma
LUSC: lung squamous cell carcinoma
PAAD: pancreatic adenocarcinoma
PCPG: pheochromocytoma and paraganglioma
PRAD: prostate adenocarcinoma
READ: rectum adenocarcinoma
STAD: stomach adenocarcinoma
TGCT: testicular germ cell tumors
THCA: thyroid carcinoma
UCS: uterine carcinosarcoma
COAD: colon adenocarcinoma
indel: insertion/deletion
ICI: immune checkpoint inhibitor
