# Supplementary material for: PSME2 offers value as a biomarker of M1 macrophage infiltration in pan-cancer and inhibits osteosarcoma malignant phenotypes
Source: Int J Biol Sci. 2024 Feb 4;20(4):1452–70. doi: 10.7150/ijbs.90226 (PMC10878157; doi:10.7150/ijbs.90226)
Supplement: Supplementary file 1 — Supplementary figures and tables. [file ijbsv20p1452s1.pdf]

Supplemental Figures For: PSME2 offers value as a biomarker of M1 macrophage infiltration in pan-cancer and inhibits malignant phenotypes of osteosarcoma

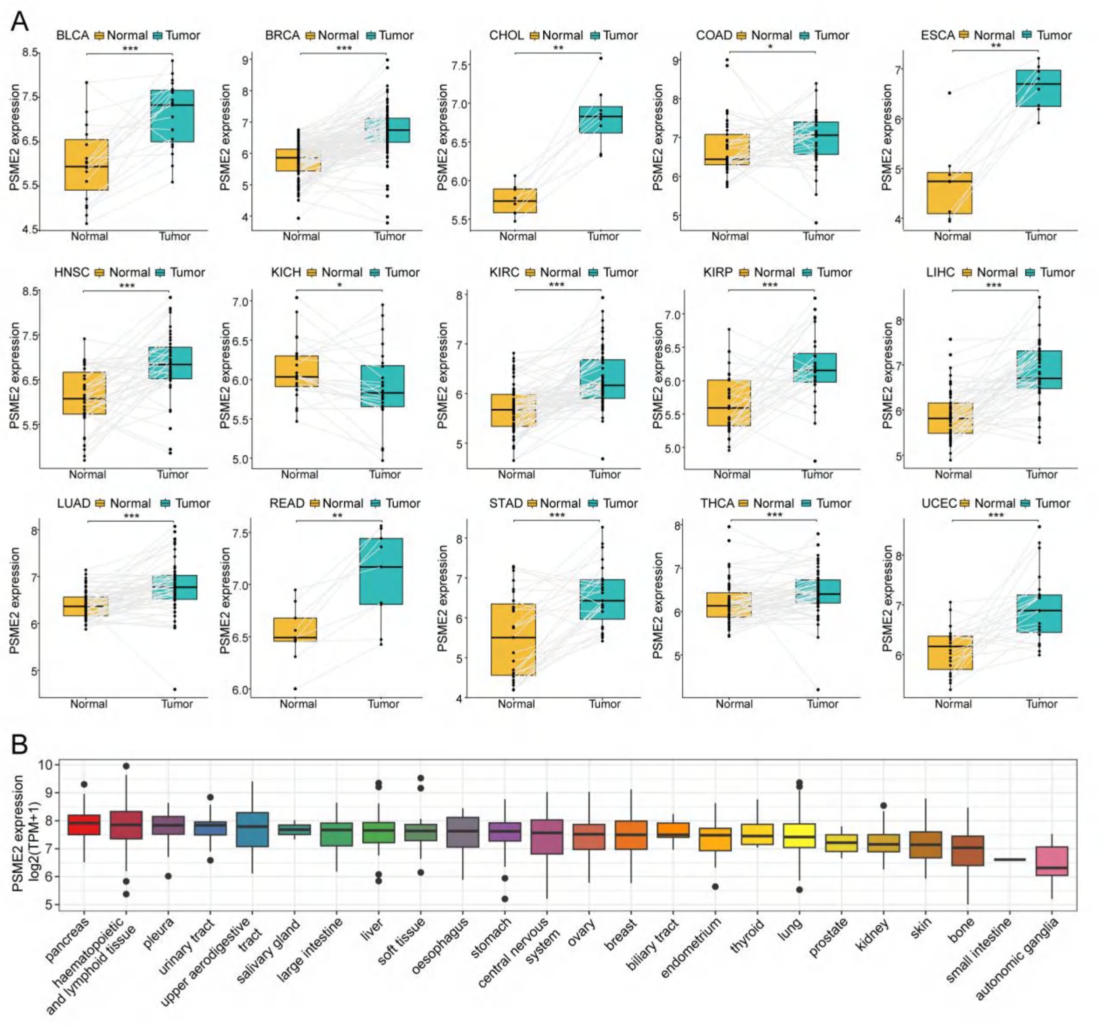

Figure S1 (A) PSME2 mRNA levels in tumors and adjacent normal tissues in the TCGA database. *P < 0.05, **P < 0.01, ***P < 0.001; ns: not significant. (B) PSME2 expression levels in tumor cell lines from CCLE database.

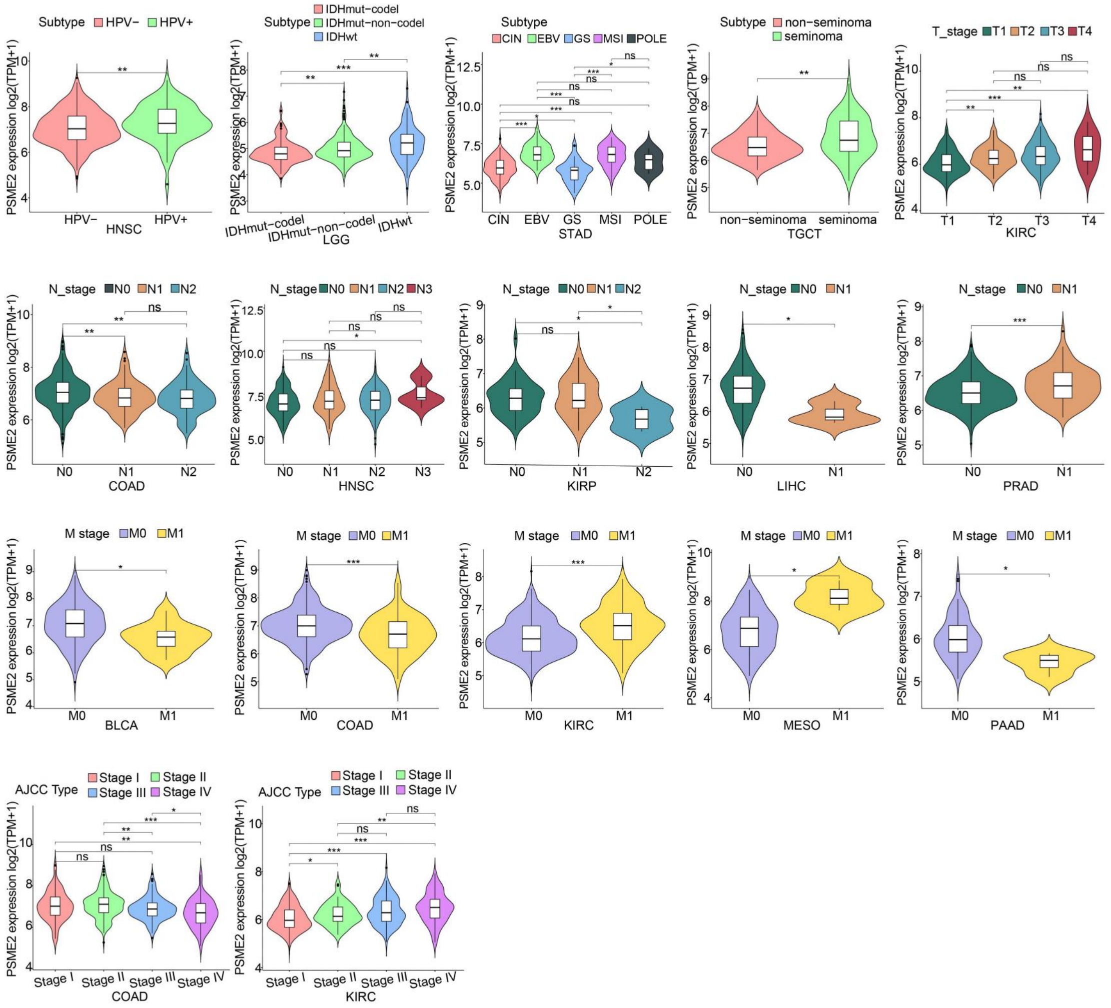

Figure S2 Pan-cancer analyses of the expression of PSME2 across different tumor stages and subtypes. *P < 0.05, **P < 0.01, ***P < 0.001; ns: not significant.

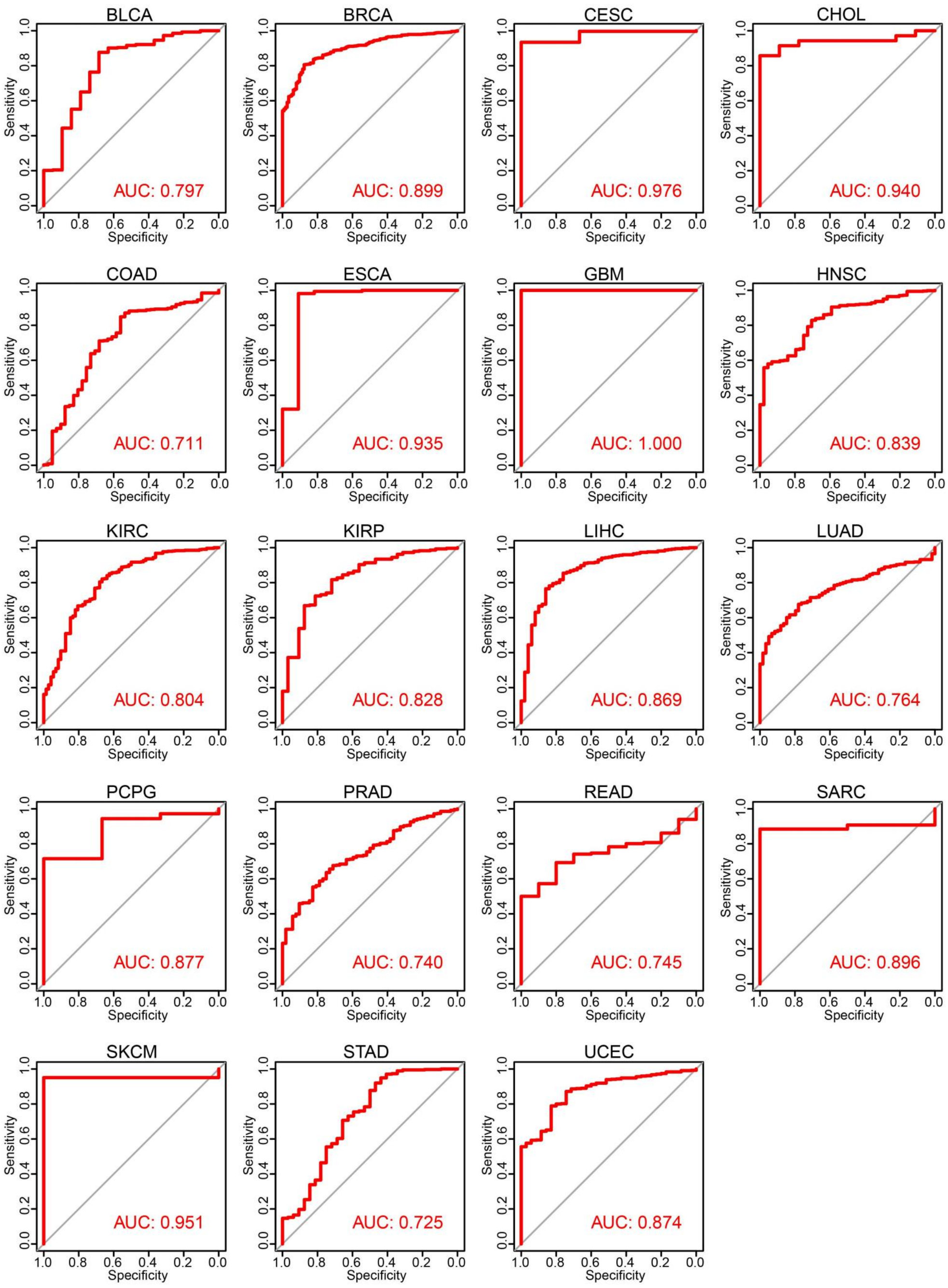

Figure S3 Pan-cancer diagnostic ROC curves.

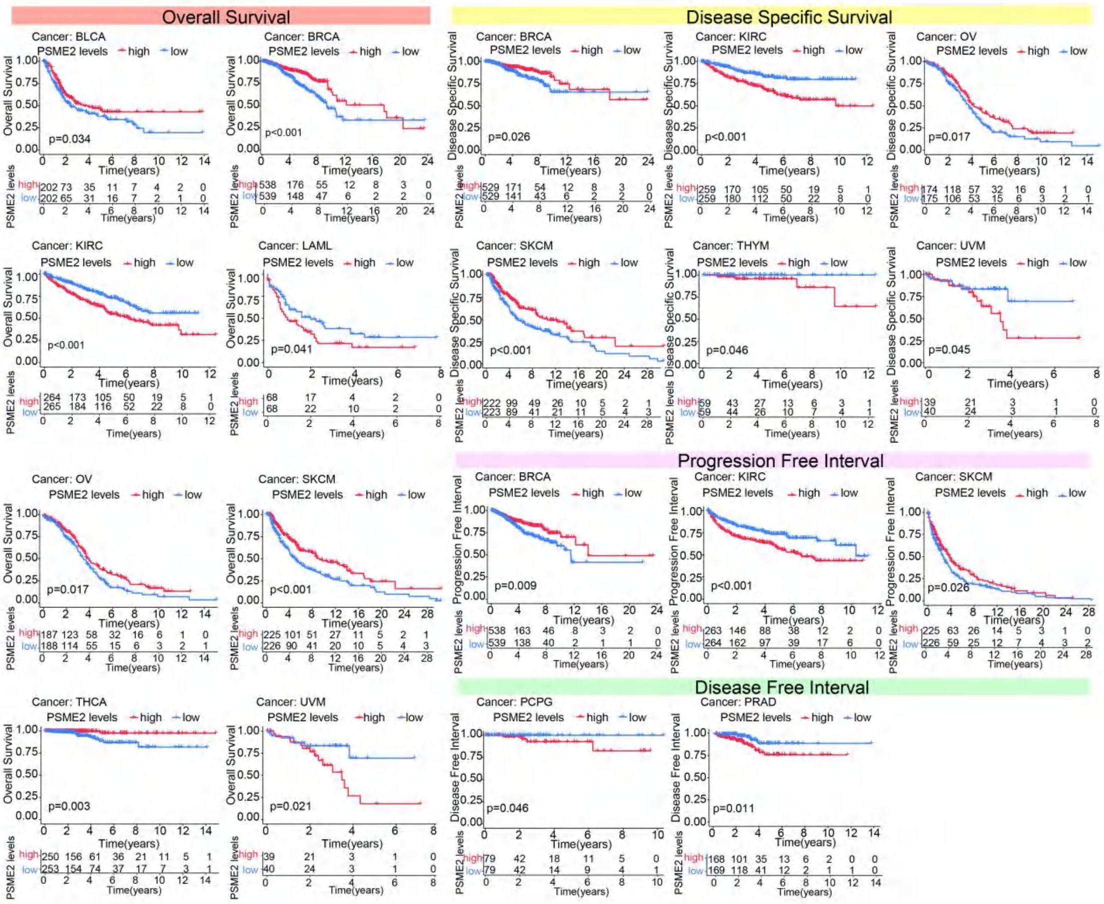

Figure S4 Kaplan-Meier curves representing associations between PSME2 levels and the OS, DSS, DFI, and PFI of patients with the indicated cancer types.

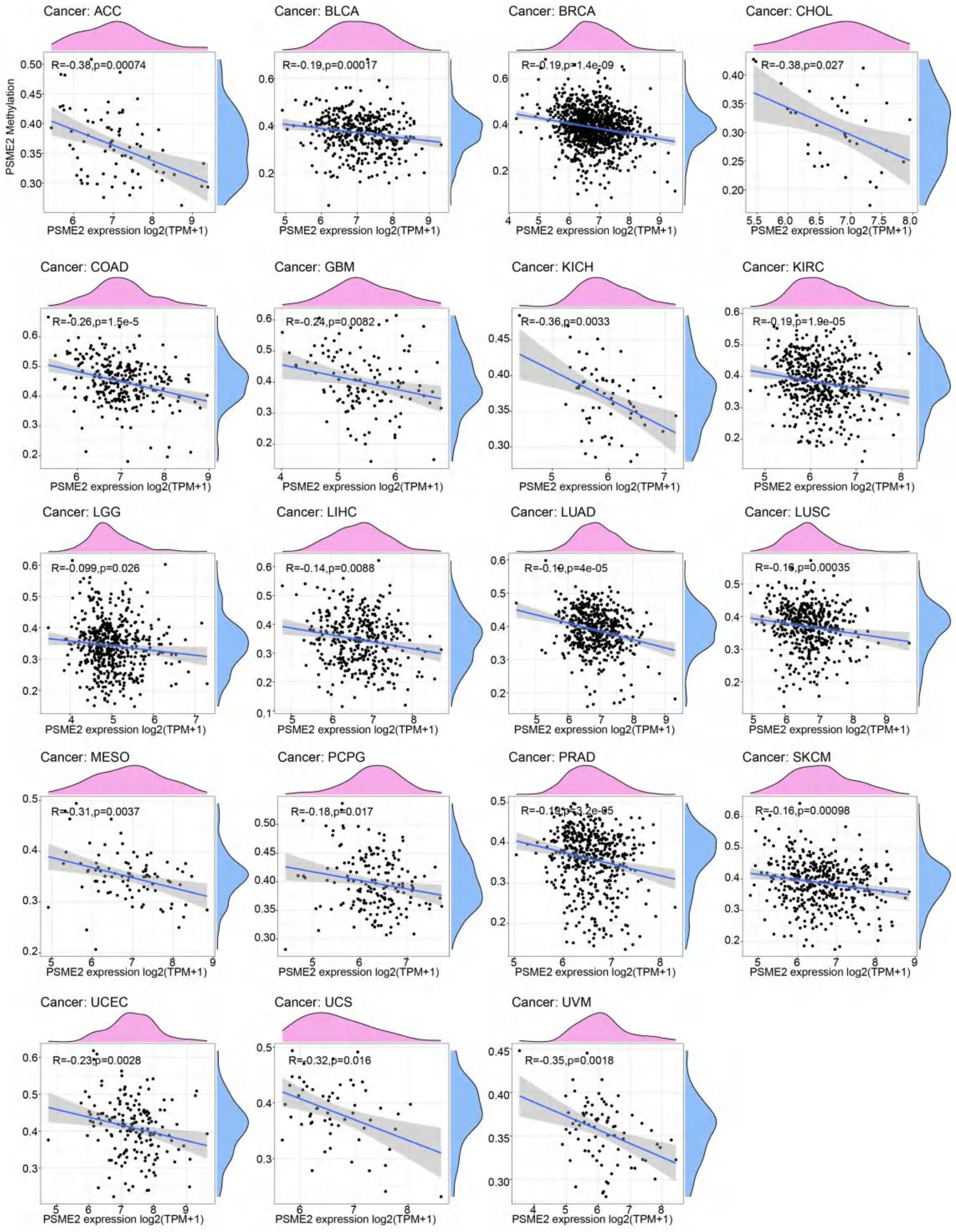

Figure S5 Pan-cancer analyses of the association between PSME2 levels and promoter methylation.

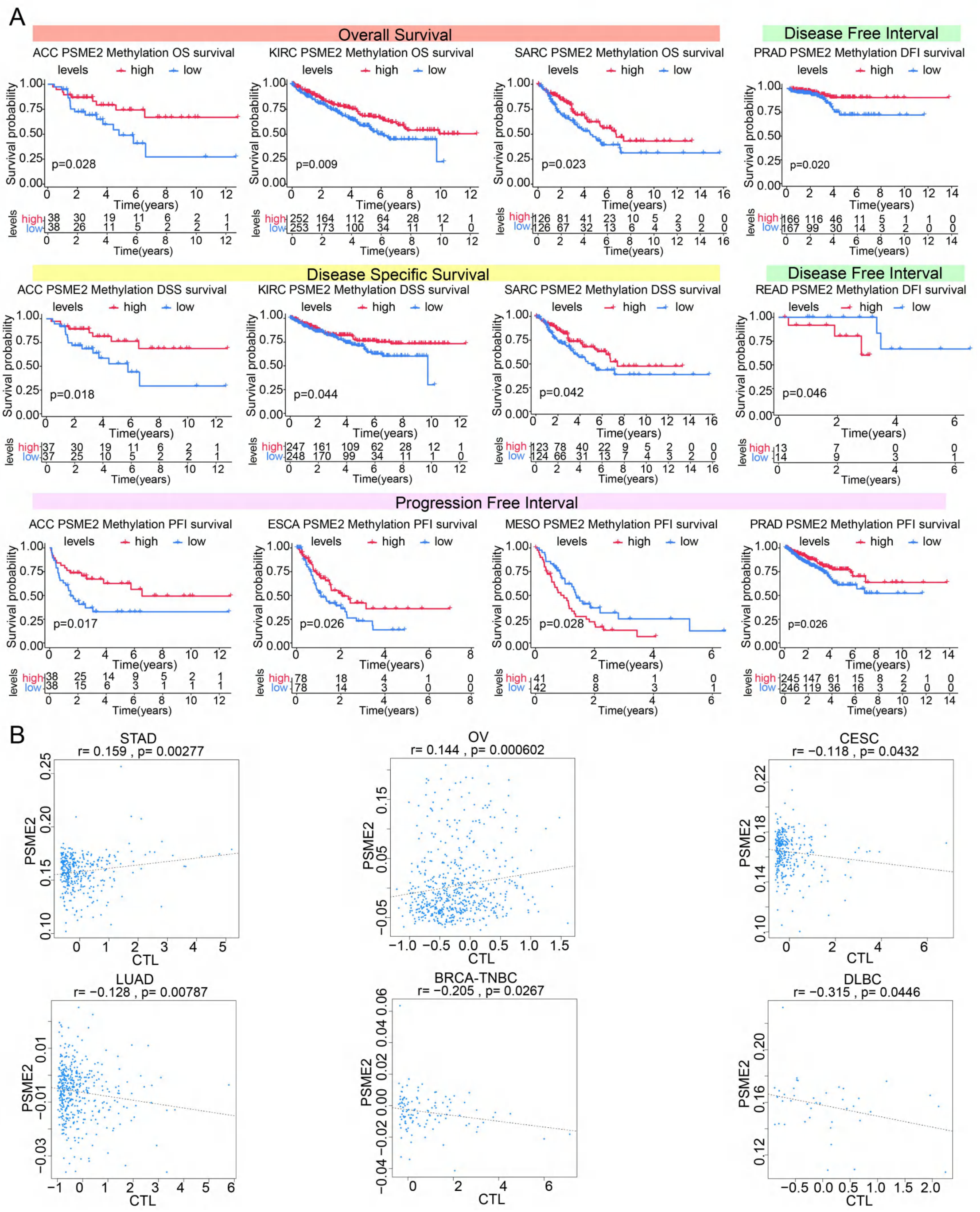

Figure S6 Correlations between PSME2 methylation and prognostic outcomes in different cancers. (A-D) Kaplan-Meier curves highlighting associations between the degree of PSME2 methylation and OS (B), DSS (C), DFI (D), and PFI (E) for the indicated cancer types. (E) Scatter plots representing relationships between levels of PSME2 methylation and markers of CTLs in specific cancer types.

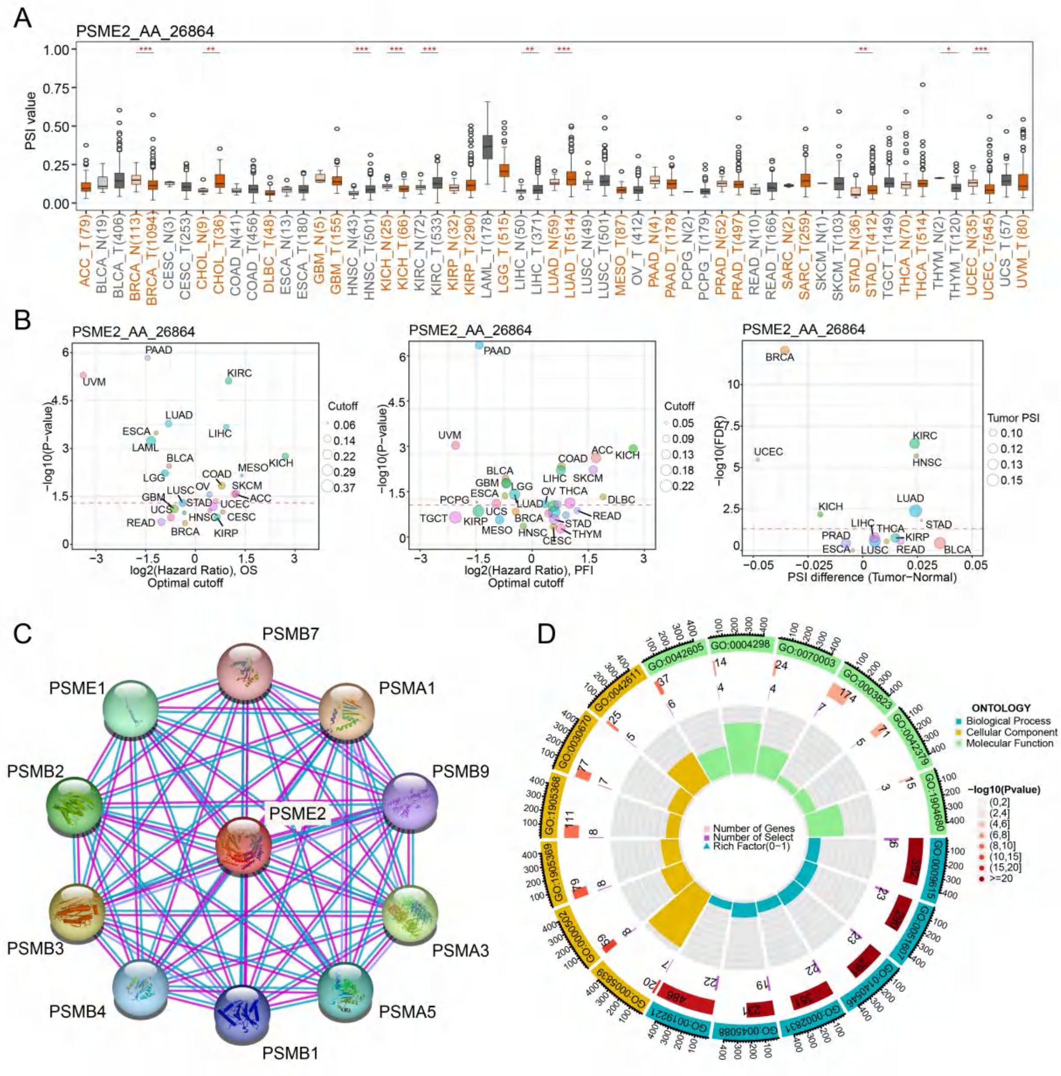

Figure S7 (A) PSME2_ AA_26864 PSI values in pan-cancer, adjacent, and normal tissues. Colored labels correspond to cancers and matching adjacent tissues. (B) Differences in PSI values between tumors and adjacent/normal tissues and the association between PSME2_AA_26864 events and prognostic outcomes. (C) A network of experimentally validated protein-protein interactions for PSME2. (D) Enriched GO pathways of the top 100 genes co-expressed with PSME2.

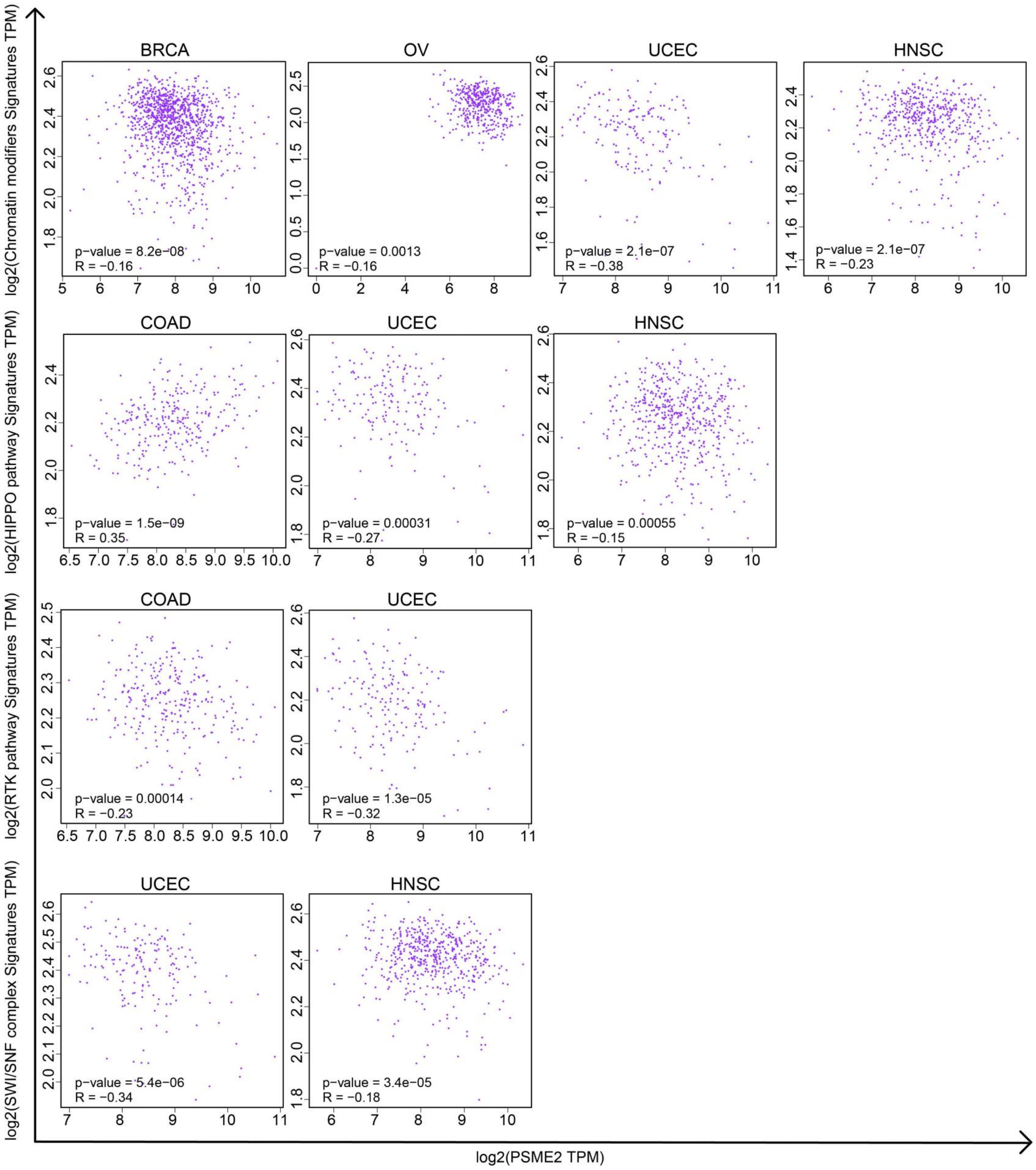

Figure S8 Correlations between PSME2 levels and oncogenic pathway signatures.

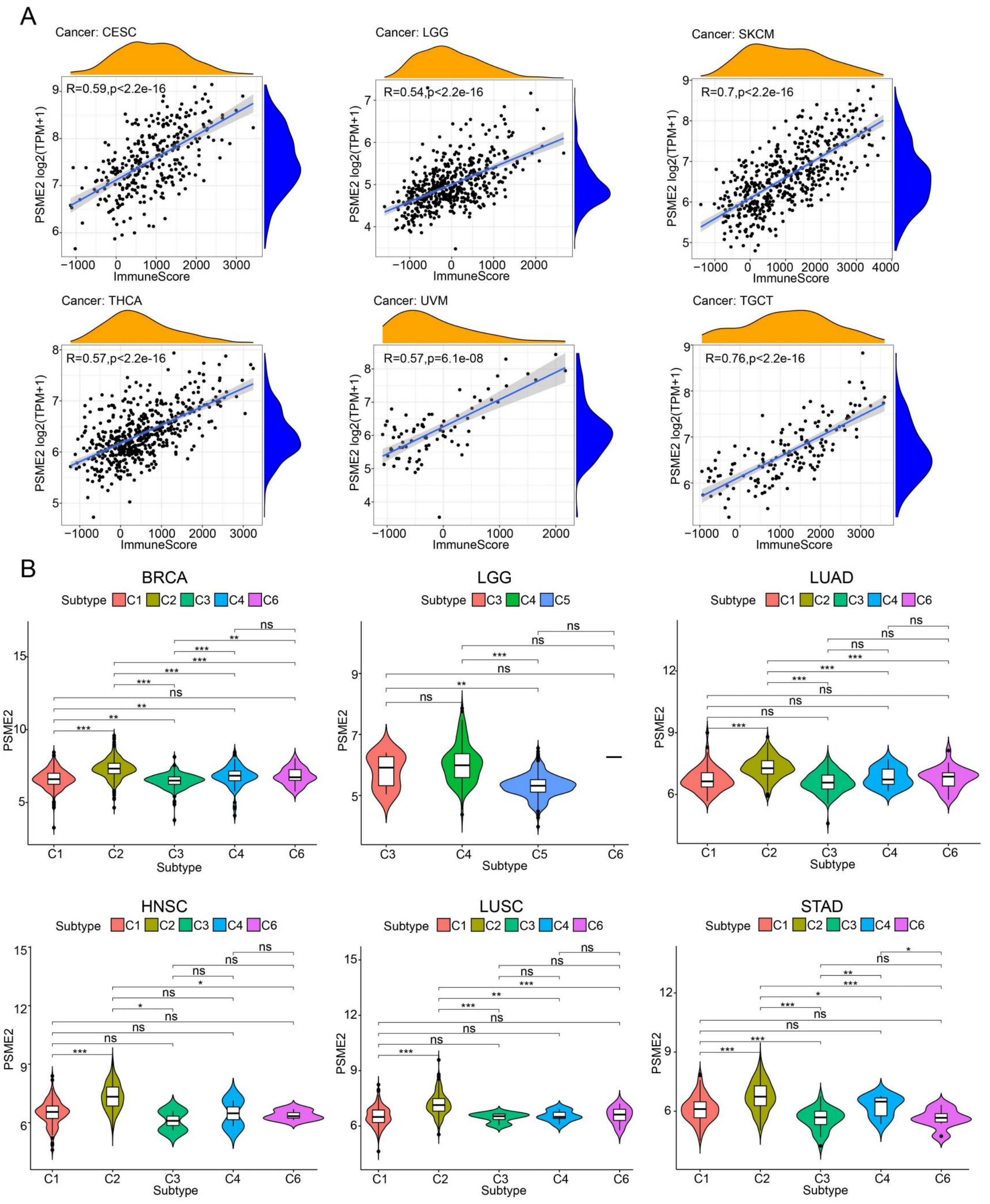

Figure S9 Associations between PSME2 expression and pan-cancer immunity. (A) Scatter plots for the 6 tumor types with the strongest correlations between PSME2 expression levels and Immune score values. (B) PSME2 expression levels in the indicated immune subtypes of BRCA, LGG, LUAD, HNSC, LUSC, and STAD tumors.

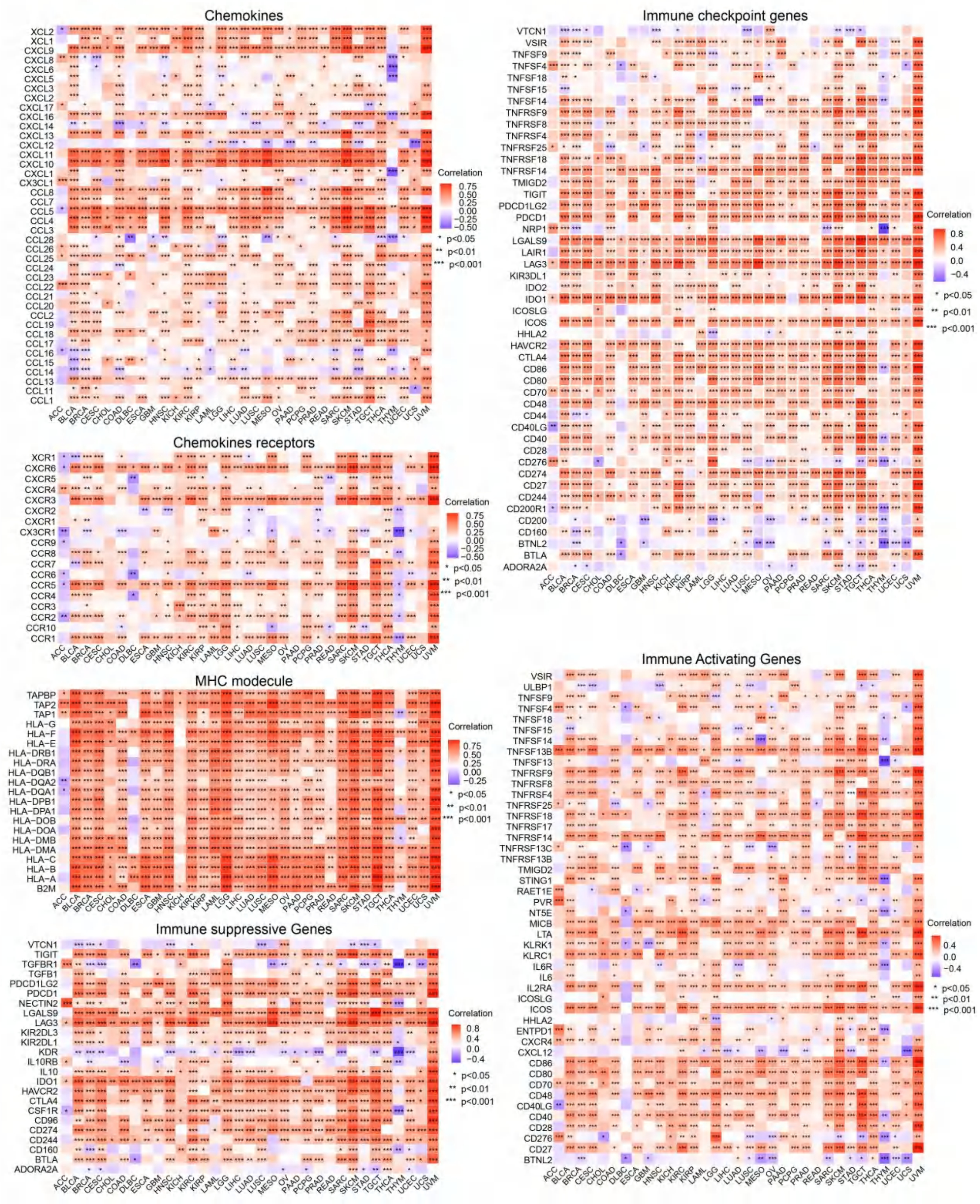

Figure S10 Correlations between the expression of PSME2 and immune-related genes. Analyzed genes included chemokines, chemokine receptors, MHC genes, immunosuppressive genes, and immune-activating genes. *P < 0.05, **P < 0.01, ***P < 0.001.

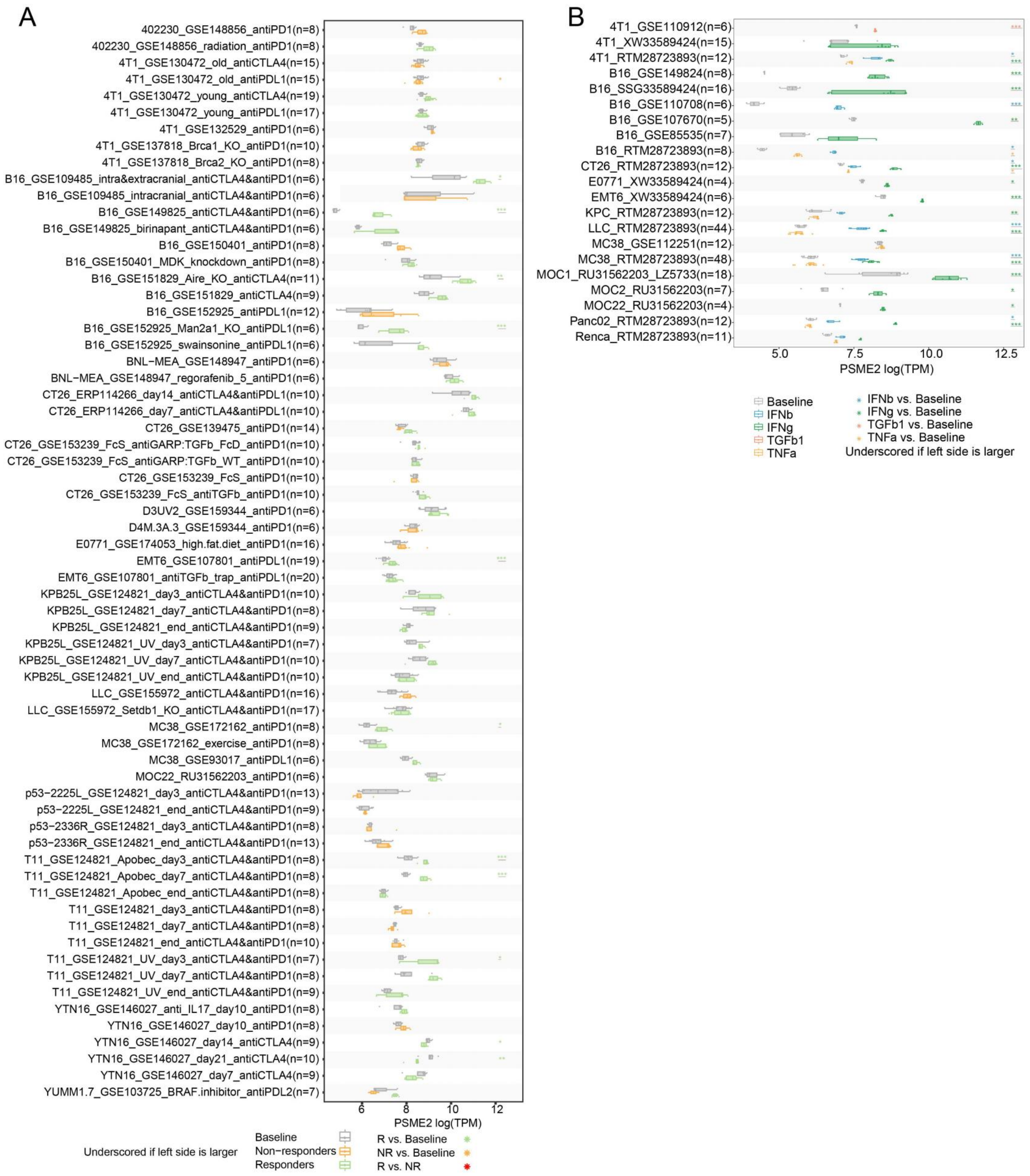

Figure S11 (A) Box plots representing PSME2 expression before and after cytokine treatment in the indicated tumor cell lines as analyzed with the TISMO tool. (B) Box plots representing the expression of PSME2 before and after treatment with ICIs (anti-PD1, anti-PDL1, and anti-CTLA4) as analyzed with the TISMO tool. *P < 0.05, **P < 0.01, ***P < 0.001.

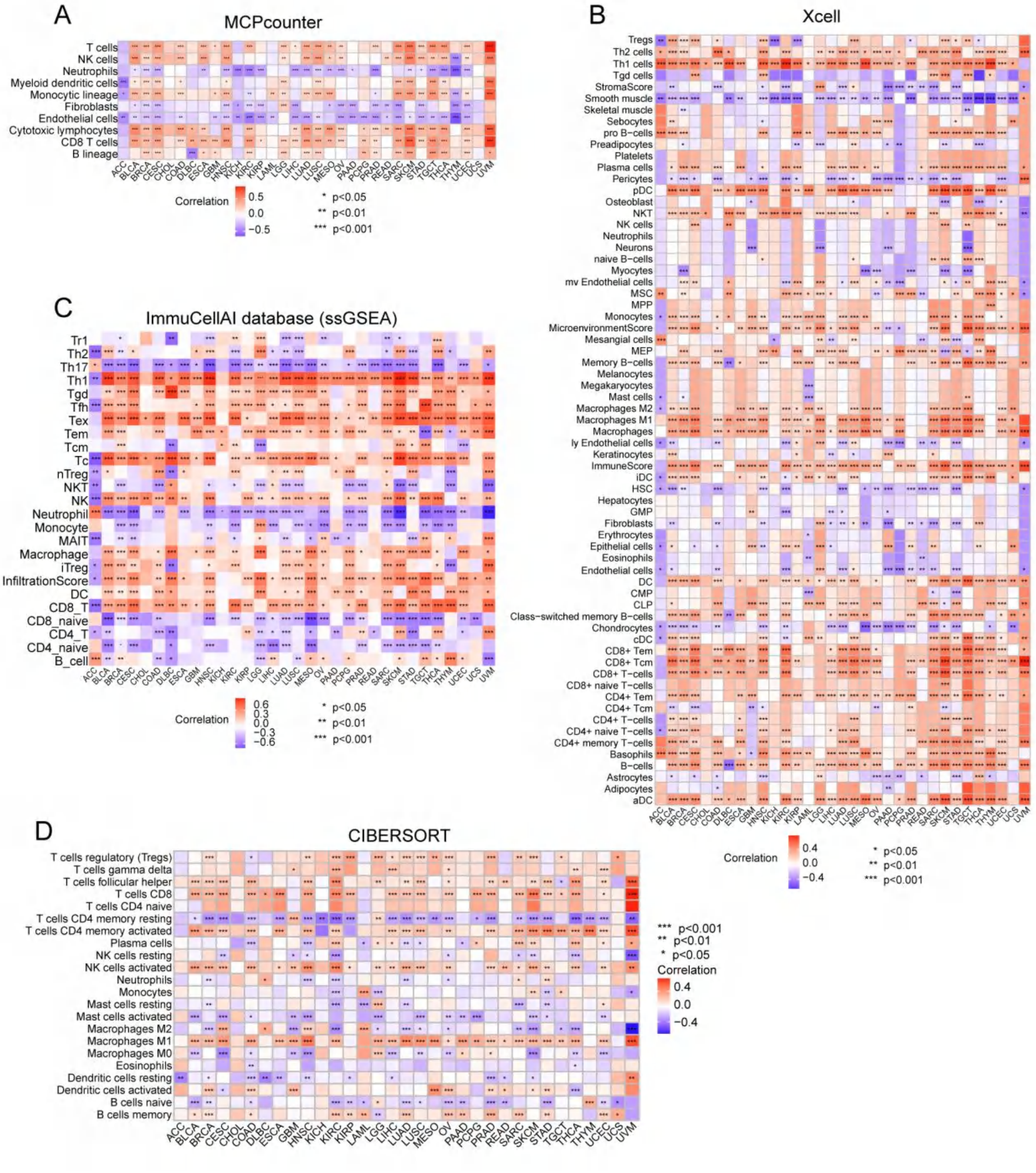

Figure S12 Correlation between PSME2 expression and the infiltration of immune cells. (A-D) Heatmap showing the association between PSME2 expression and the infiltration of immune cells in pan-cancer generated by using MCPCOUNTER algorithm (A), Xcell algorithm (B), ssGSEA algorithm from the ImmuCellAI database (C), and CIBERSORT algorithm (D).

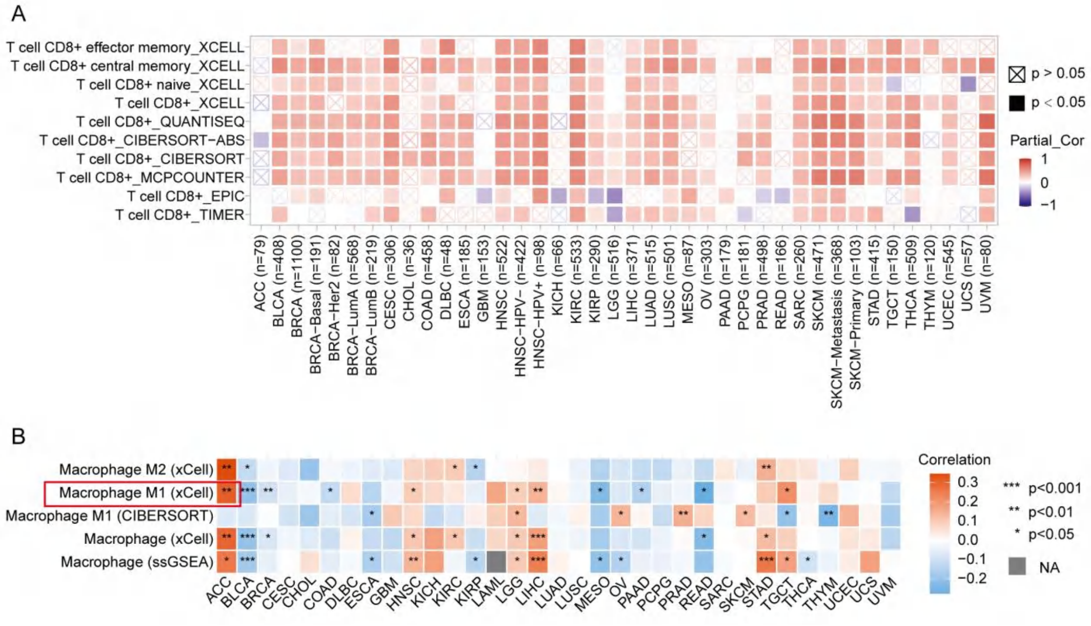

Figure S13 (A) TIMER2.0 was used to calculate the association between PSME2 expression and the infiltration of CD8+ T-cell infiltration in different cancers using multiple algorithms. (B) The association between PSME2 promoter methylation and M1 macrophage infiltration.

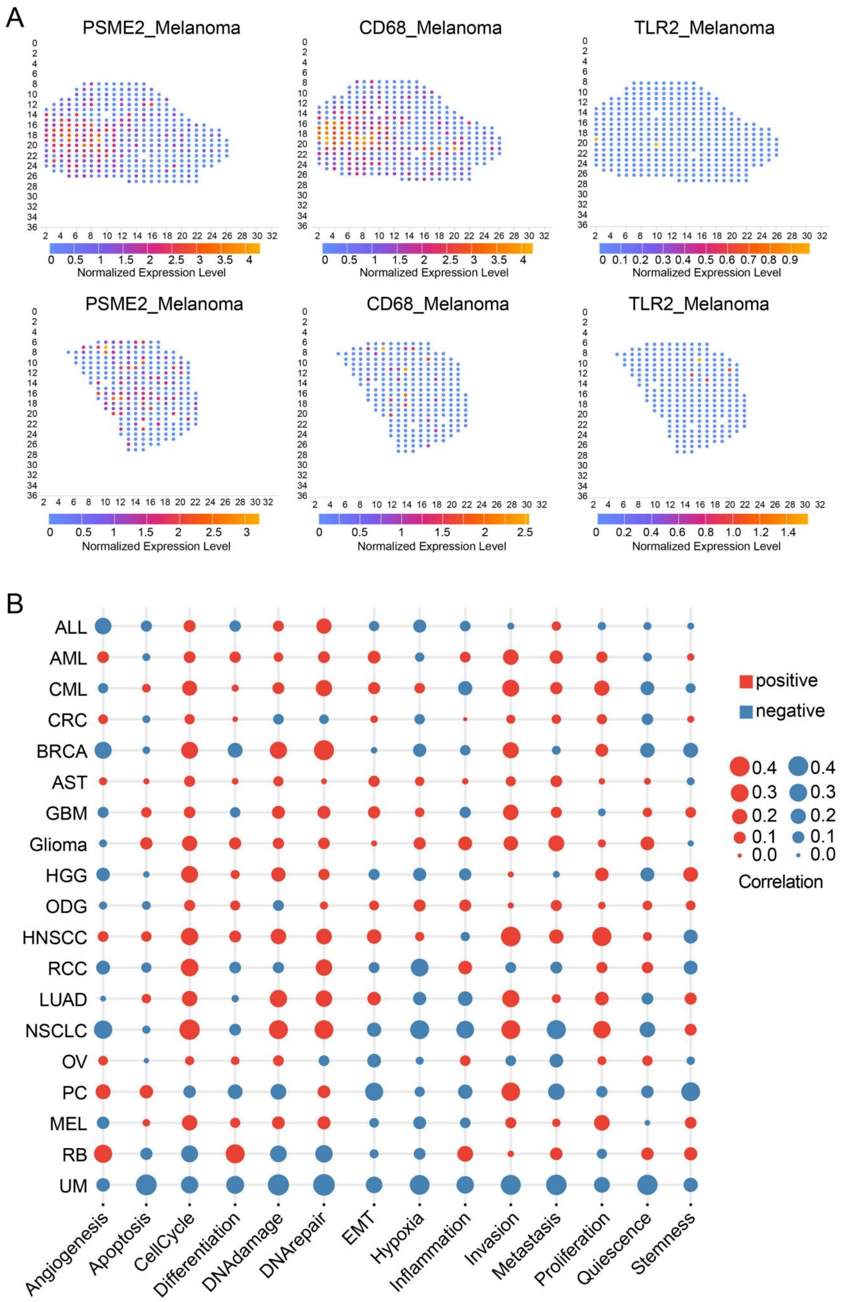

A
PSME2_Melanoma
CD68_Melanoma
TLR2_Melanoma
Normalized Expression Level
PSME2_Melanoma
CD68_Melanoma
TLR2_Melanoma
Normalized Expression Level
B
ALL
AML
CML
CRC
BRCA
AST
GBM
Glioma
HGG
ODG
HNSCC
RCC
LUAD
NSCLC
OV
PC
MEL
RB
UM
Angiogenesis
Apoptosis
CellCycle
Differentiation
DNAdamage
DNArepair
EMT
Hypoxia
Inflammation
Invasion
Metastasis
Proliferation
Quiescence
Stemness
positive
negative
0.4
0.3
0.2
0.1
0.0
Correlation

Figure S14 (A) Spatial transcriptional sections presenting the spatial relationships between PSME2, CD68, and TLR2 expression in melanoma. Dot colors are indicative of gene expression levels. (B) Correlations between PSME2 expression and 14 cancer functional states as analyzed with the CancerSEA single-cell sequencing dataset. Abbreviations: ALL: acute lymphoblastic leukemia; AML, acute myeloid leukemia; CML, chronic myelogenous leukemia; CRC, colorectal cancer; AST, astrocytoma; HGG, high-grade glioma; ODG, oligodendroglioma; HNSCC, head and neck cancer; RCC, renal cell carcinoma; NSCLC, non-small cell lung cancer; PC, prostate cancer; MEL, melanoma; RB, retinoblastoma; UM, uveal melanoma.

Supplementary Material 1. The FASTA sequence used for PSME2 protein homology modeling.

```
>CAG46543.1 PSME2 [Homo sapiens]
MAKPCGVRLSGEVRKQVEVFRQNLFQEAEEFLYRFLPQKIIYLNQLLQEDSLNVADL
TSLRAPLDIPIPDPPPKDDEMETDKQEKKEVPKCGFLPGNEKVLSLLALVKPEVWTLK
EKCILVITWIQHLIPKIEDGNDFGVAIQEKVLERVNAVKTKVEAFQTTISKYFSERGDA
VAKASKETHVMDYRALVHERDEAAYVELRAMVLDLRAFYAELYHIISSNLEKIVNP
KGEEKPSMY
```

Supplementary Material 2.

Experimental methods

Multiple Fluorescence Staining of Pan-Cancer Tissue Chip

Multiple fluorescence staining was performed on pan-cancer paraffin sections to validate the M1 macrophage biomarker potential of PSME2. The sections in this study contained 10 cancer types. These sections were deparaffinized and blocked with 5% bovine serum albumin (BSA). Subsequently, they were incubated sequentially with the primary antibodies, PSME2 (mouse, 1:200, ab183727), TLR2 (rabbit, 1:10, ab9100). After primary antibody labeling, the sections were incubated by secondary antibody (BA1031, BA1105, Boster, Wuhan, China). Next, 4′,6-diamidino-2-phenylindole dihydrochloride (DAPI) counterstaining of nuclei, the antifade mounting medium was applied, and a confocal microscope (Pannoramic MIDI, 3DHistech, Hungary) was used to obtain multispectral images of the stained sections. For fluorescence spectra, the excitation wavelength and emission wavelength for different fluorescence dyes are listed respectively as follows: DAPI (blue, 330–380 and 420 nm), CY3 (red, 510–560 and 590 nm), and FITC (green, 465–495 and 515–555 nm). Finally, Caseviewer (C.V 2.4) image analysis software was used to display the cells with positive staining at single-cell levels in the multispectral images.

Cell lines and culture

Human osteosarcoma cell lines (HOS and U2OS) were purchased from the American Type Culture Collection (ATCC). Cells were maintained in DMEM/F12 containing 10% fetal bovine serum and 1% penicillin/ streptomycin (Invitrogen, USA) in a humidified incubator at 37 °C with 5% CO_2.

Cell transfection

Cells were cultured in 6-well plates until 40% confluent after which they were transduced with lentiviral (Genechem) for 24 h and selected with puromycin (3 mg/mL) for 24 hours. Transfections with lentivirus were performed with a multiplicity of infection (MOI)=20 in HOS cell line and a MOI=10 in U2OS cell line. Stability of expression was determined.

RNA isolation and qRT-PCR

Total RNA was extracted from transfected cells with TRIzol (Thermo Fisher Scientific) according to the provided directions. Gene expression relative to 18S rRNA was measured by qRT-PCR using an IQ5 Multicolour Real-Time PCR Detection system (Bio-Rad Laboratories, CA, USA).

PCR detection included reaction at 95 °C (3 min), 95 °C (15 s) for 40 cycles, 56.5 °C (15 s), and 72 °C (30 s). The sequences of human PSME2 primer were (forward) 5'-TCCAAGGAGACTCATGTAATGGATT-3' and (reverse) 5'-CCCCATAGGCTGCCTCATC-3'. The sequences of human 18S primer were (forward) 5'-AAACGGCTACCACATCCA-3' and (reverse) 5'-CACCACTTGCCCCTCCA-3'.Relative expression was determined by the $2^{-\Delta\Delta CT}$ method [1].

Western blotting

Proteins were extracted from cells in RIPA buffer with a protease and phosphatase inhibitor cocktail (Keygen, Nanjing, China). After determination of concentrations using BCA, the proteins were separated on SDS-PAGE and transferred to nitrocellulose. The blots were treated with anti-PSME2 (1:3000) (ab183727, Abcam, UK) and anti-GAPDH (1:5000) (ab181602, Abcam, UK) antibodies and the bands were visualized using enhanced chemiluminescence. The loading control was GAPDH.

Cell proliferation assays

CCK-8 assays were performed by seeding and maintaining 6000 cells in 96-well plates for 1, 2 or 3 days. Fresh medium was replaced with 100μL, and CCK-8 (10μL, Sigma) was added. Cells were then incubated for 2 hours for OD 450 measurement. Cells were fixed and permeabilized after EdU was added to culture medium at 10 μM and incubated for 2h. Click-iT reaction was performed following the manufacturer's manual (ThermoFisher Scientific). As a final step, nuclear staining was carried out using DAPI, and images were obtained using a fluorescence microscope (ThermoFishr Scientific). In colony formation assays, 600 cells were seeded and cultured for 10 days in 6-well plates. Next, they were washed with PBS and fixed with 4% paraformaldehyde for 30 minutes. Cells were stained with 0.5% crystal violet for 1 hr at room temperature, and colonies were counted with clone-counter program.

The viability of irinotecan alone or in combination with paclitaxel in osteosarcoma cells was analysed by the CCK8 assay. Briefly, HOS or U2OS cells underwent seeding process in a final volume of 100 μL in a 96 well plate at 3000 cells/well density and cultured for 24 hours. After adding different concentrations of irinotecan and/or paclitaxel, the cells were further cultured for another 48 hours. Then, we dropped CCK-8 regent (10μL, Sigma) in every well, and the culture plate underwent incubation as shielded from light for 2 hours at 37°C. We ascertained optical

density (OD) value at a wavelength of 450 nm. Using the median-effect model of Chou-Talalay and with CompuSyn software, we ascertained the combination index (CI) [2]. CI < 1 indicates synergism. Each experiment was carried out in triplicate.

Wound-healing assay

10^6 cells were seeded per well in a six-well plate after being trypsinized. Micropipettes were scraped with sterile tips after overnight incubation. We photographed the initial gap width and the residual gap width 24 hours after scratching.

Transwell assays

Migration assays were carried out in Transwell chambers 8 mm in diameter, Corning. For the invasion assay, growth factor-reduced Matrigel (354234; Corning) was precoated on the chambers before the migration assays. A suspension of osteosarcoma cells in 200 μL serum-free DMEM medium was added to the upper chamber, while 10% fetal bovine serum was added to the bottom chamber. After 24 hours of cultivation, cells in the chamber were fixed with 4% paraformaldehyde for 30 minutes and stained with 0.1% crystal violet. We viewed cells under an inverted light microscope in three random fields of view that had migrated or invaded to the lower surface.

In vivo growth

Animal experiments were carried out in accordance with the Guide for the Care and Use of Laboratory Animals (Ministry of Science and Technology of China, 2006), and were approved by the animal ethics committee of Shanxi Medical University (approval number DW2022054). Five-week-old BALB/c nude mice were obtained from Charles River (China). Randomly selected mice received subcutaneous injections of 10^6 lentivirus-transfected HOS cells in 200 μL Matrigel (n = 6 per group). Tumor growth was assessed each day. The mice were euthanized after five weeks, and the tumors were removed and measured [3].

Immunohistochemistry

Tissue samples from xenograft tumors were fixed with 4% paraformaldehyde for 14 h, paraffin-embedded, and sectioned. After dewaxing, rehydration, and blocking (5% BSA), the sections were treated with Ki67 (1:1000) (ab15580, Abcam, UK) or PCNA (1:10000) (2586, Cell Signaling Technology, USA) overnight at 4°C.

1. Livak KJ, Schmittgen TD. Analysis of relative gene expression data using real-time

quantitative PCR and the 2(-Delta Delta C(T)) Method. *METHODS.* 2001;25(4):402-408.

2. Chou TC. Drug combination studies and their synergy quantification using the Chou-Talalay method. *Cancer Res.* 2010;70(2):440-446.
3. Naito S, von Eschenbach AC, Giavazzi R, Fidler IJ. Growth and metastasis of tumor cells isolated from a human renal cell carcinoma implanted into different organs of nude mice. *Cancer Res.* 1986;46(8):4109-4115.

Table S1. List of abbreviations.

Abbreviations	Full name
adrenocortical carcinoma	ACC
bladder urothelial carcinoma	BLCA
breast invasive carcinoma	BRCA
Clear cell renal cell carcinoma	ccRCC
cervical squamous cell carcinoma and endocervical adenocarcinoma	CESC
cholangiocarcinoma	CHOL
colon adenocarcinoma	COAD
disease-free interval	DFI
lymphoid neoplasm diffuse large B-cell lymphoma	DLBC
disease-specific survival	DSS
esophageal carcinoma	ESCA
glioblastoma multiforme	GBM
head and neck squamous cell carcinoma	HNSC
kidney chromophobe	KICH
kidney renal clear cell carcinoma	KIRC
kidney renal papillary cell carcinoma	KIRP
brain lower-grade glioma	LGG
liver hepatocellular carcinoma	LIHC
lung adenocarcinoma	LUAD
lung squamous cell carcinoma	LUSC
mesothelioma	MESO
overall survival	OS
ovarian serous cystadenocarcinoma	OV
pancreatic adenocarcinoma	PAAD
pheochromocytoma and paraganglioma	PCPG
progression-free interval	PFI
prostate adenocarcinoma	PRAD
rectum adenocarcinoma	READ
sarcoma	SARC
skin cutaneous melanoma	SKCM
stomach adenocarcinoma	STAD
testicular germ cell tumors	TGCT
thyroid carcinoma	THCA
uterine corpus endometrial carcinoma	UCEC
uterine carcinosarcoma	UCS
Thymoma	THYM
Uveal Melanoma	UVM
acute myeloid leukemia	LAML
Proteasome Activator Subunit 2	PSME2
Proteasome Activator 28	PA28
The Cancer Genome Atlas	TCGA

Abbreviations	Full name
Genotype-Tissue Expression	GTEx
tumor microenvironment	TME
major histocompatibility complex	MHC
endemic Burkitt's lymphoma	eBL
gastric adenocarcinoma	GA
chloride ion intracellular channel 1	CLIC1
cytotoxic T lymphocytes	CTLs
simple-nucleotide variation	SNV
transcripts per million	TPM
The Clinical Proteomic Tumor Analysis Consortium	CPTAC
The University of Alabama at Birmingham Cancer data analysis Portal	UALCAN
receiver operating characteristic curves	ROC
confidence intervals	CI
hazard ratio	HR
Tumor Immune Dysfunction and Exclusion	TIDE
copy number variations	CNV
tumor mutation burden	TMB
microsatellite instability	MSI
homologous recombination deficiency	HRD
mismatching repair	MMR
homologous recombination repair	HHR
Gene Expression Profiling Interactive Analysis	GEPIA
differentially methylated probes-based stemness index	DMPsi
N1-methyladenosine	m1A
5-methylcytosine	m5C
N6-methyladenosine	m6A
the percent spliced-in	PSI
protein–protein interaction	PPI
Gene Ontology	GO
false discovery rate	FDR
Gene Set Enrichment Analysis	GSEA
Estimation of Stromal and Immune Cells in Malignant Tumor Tissues Using Expression Data	ESTIMATE
breast cancer	BC
the Tumor Immune Single-cell Hub	TISCH
bovine serum albumin	BSA
tyramide signal amplification	TSA
4′,6- diamidino-2-phenylindole dihydrochloride	DAPI
mechanisms of action	MoA
50% growth inhibition	GI50
Developmental Therapeutics Program	DTP
National Cancer Institute	NCI

**Abbreviations**	**Full name**
One-way analysis of variance	ANOVA
MHC proteins	pMHCs
area under the curve	AUC
immunocheckpoint inhibitor	ICIs
human leukocyte antigens	HLA
Janus kinase	JAK
signal transducer and activator of transcription 1	STAT1

Table S2. 51 PSME2 clinical-relevant AS events on OncoSplicing.

Gene_Symbol	Splice_Type	Splice_Event	Project
PSME2	AA	PSME2_AA_26862	SpliceSeq
PSME2	AA	PSME2_AA_26863	SpliceSeq
PSME2	AA	PSME2_AA_26864	SpliceSeq
PSME2	ES	PSME2_ES_195219	SpliceSeq
PSME2	ES	PSME2_ES_26865	SpliceSeq
PSME2	ES	PSME2_ES_26866	SpliceSeq
PSME2	A3	alt_3prime_72269	SplAdder
PSME2	A3	alt_3prime_72270	SplAdder
PSME2	A3	alt_3prime_72272	SplAdder
PSME2	A3	alt_3prime_72277	SplAdder
PSME2	A3	alt_3prime_72279	SplAdder
PSME2	A3	alt_3prime_72283	SplAdder
PSME2	A3	alt_3prime_72289	SplAdder
PSME2	A3	alt_3prime_72294	SplAdder
PSME2	A3	alt_3prime_72296	SplAdder
PSME2	A3	alt_3prime_72297	SplAdder
PSME2	A3	alt_3prime_72300	SplAdder
PSME2	A3	alt_3prime_72302	SplAdder
PSME2	A3	alt_3prime_72304	SplAdder
PSME2	A3	alt_3prime_72307	SplAdder
PSME2	A5	alt_5prime_53528	SplAdder
PSME2	A5	alt_5prime_53534	SplAdder
PSME2	ES	exon_skip_111910	SplAdder
PSME2	ES	exon_skip_111914	SplAdder
PSME2	ES	exon_skip_111921	SplAdder
PSME2	ES	exon_skip_111929	SplAdder
PSME2	ES	exon_skip_111931	SplAdder
PSME2	ES	exon_skip_111955	SplAdder
PSME2	ES	exon_skip_111957	SplAdder
PSME2	ES	exon_skip_111958	SplAdder
PSME2	ES	exon_skip_111959	SplAdder
PSME2	IR	intron_retention_40243	SplAdder
PSME2	IR	intron_retention_40247	SplAdder
PSME2	IR	intron_retention_40253	SplAdder
PSME2	IR	intron_retention_40254	SplAdder
PSME2	IR	intron_retention_40260	SplAdder
PSME2	IR	intron_retention_40261	SplAdder
PSME2	IR	intron_retention_40262	SplAdder
PSME2	IR	intron_retention_40263	SplAdder
PSME2	IR	intron_retention_40267	SplAdder
PSME2	IR	intron_retention_40268	SplAdder
PSME2	IR	intron_retention_40275	SplAdder

Gene_Symbol	Splice_Type	Splice_Event	Project
PSME2	IR	intron_retention_40276	SpIAdder
PSME2	IR	intron_retention_40280	SpIAdder
PSME2	IR	intron_retention_40282	SpIAdder
PSME2	IR	intron_retention_40283	SpIAdder
PSME2	IR	intron_retention_40286	SpIAdder
PSME2	IR	intron_retention_40288	SpIAdder
PSME2	IR	intron_retention_40290	SpIAdder
PSME2	IR	intron_retention_40291	SpIAdder

Table S3. GO pathways enriched by the top 100 PSME2 co-expressed genes identified on GEPIA2.0.

ONTOLOGY	ID	Description	Gene Ratio	BgRatio	pvalue	p.adjust	qvalue	geneID	Count
BP	GO:0009615	response to virus	26/82	392/18800	4.95E-24	7.80E-21	5.71E-21	IRF1/TRIM21/STAT1/NMI/IRF9/IFI27/GBP1/MOV10/PARP9/ISG15/IRF7/BST2/AIM2/ISG20/IFNG/CXCL10/IFI44/RTP4/CCL5/ZBP1/CXCL9/NLRC5/OASL/IRF3/OAS2/OAS1	26
BP	GO:0051607	defense response to virus	23/82	290/18800	5.21E-23	2.96E-20	2.17E-20	IRF1/TRIM21/STAT1/IRF9/IFI27/GBP1/MOV10/PARP9/ISG15/IRF7/BST2/AIM2/ISG20/IFNG/CXCL10/RTP4/ZBP1/CXCL9/NLRC5/OASL/IRF3/OAS2/OAS1	23
BP	GO:0140546	defense response to symbiont	23/82	291/18800	5.64E-23	2.96E-20	2.17E-20	IRF1/TRIM21/STAT1/IRF9/IFI27/GBP1/MOV10/PARP9/ISG15/IRF7/BST2/AIM2/ISG20/IFNG/CXCL10/RTP4/ZBP1/CXCL9/NLRC5/OASL/IRF3/OAS2/OAS1	23
BP	GO:0002831	regulation of response to biotic stimulus	22/82	351/18800	8.80E-20	3.46E-17	2.53E-17	IFI35/IRF1/TRIM21/HLA-F/HLA-B/STAT1/NMI/LAG3/GBP5/PARP9/ISG15/IRF7/AIM2/CCL5/HLA-A/ZBP1/NLRC5/OASL/RNF31/IRF3/OAS1/SLAMF8	22
BP	GO:0045088	regulation of innate immune response	19/82	231/18800	2.36E-19	7.43E-17	5.44E-17	IFI35/IRF1/TRIM21/HLA-F/HLA-B/NMI/LAG3/GBP5/PARP9/ISG15/IRF7/AIM2/CCL5/HLA-A/ZBP1/NLRC5/IRF3/OAS1/SLAMF8	19
BP	GO:0019221	cytokine-mediated signaling pathway	22/82	486/18800	8.98E-17	2.36E-14	1.72E-14	IRF1/STAT1/NMI/IFI27/PARP9/ISG15/IRF7/AIM2/IFNG/IL15RA/CXCL10/CXCR6/CCL5/ZBP1/CXCL9/NLRC5/OASL/CXCL11/IRF3/OAS2/OAS1/CXCR3	22
CC	GO:0005839	proteasome core complex	7/82	20/19594	1.28E-12	1.91E-10	1.45E-10	PSMB9/PSMB8/PSMB10/PSMA3/PSMA5/PSMA4/PSMA6	7

ONTOLOGY	ID	Description	GeneRatio	BgRatio	pvalue	p.adjust	qvalue	geneID	Count
CC	GO:0000502	proteasome complex	8/82	59/19594	1.24E-10	9.22E-09	6.97E-09	PSME1/PSMB9/PSMB8/PSMB10/PSMA3/PSMA5/PSMA4/PSMA6	8
CC	GO:1905369	endopeptidase complex	8/82	79/19594	1.36E-09	6.76E-08	5.11E-08	PSME1/PSMB9/PSMB8/PSMB10/PSMA3/PSMA5/PSMA4/PSMA6	8
CC	GO:1905368	peptidase complex	8/82	111/19594	2.07E-08	7.70E-07	5.82E-07	PSME1/PSMB9/PSMB8/PSMB10/PSMA3/PSMA5/PSMA4/PSMA6	8
CC	GO:0030670	phagocytic vesicle membrane	7/82	77/19594	3.29E-08	9.80E-07	7.41E-07	TAP1/TAP2/HLA-F/HLA-B/HLA-C/HLA-A/B2M	7
CC	GO:0042611	MHC protein complex	5/82	25/19594	5.64E-08	1.40E-06	1.06E-06	HLA-F/HLA-B/HLA-C/HLA-A/B2M	5
MF	GO:0042605	peptide antigen binding	6/82	37/18410	1.35E-08	2.92E-06	2.33E-06	TAP1/TAP2/HLA-F/HLA-B/HLA-C/HLA-A	6
MF	GO:0004298	threonine-type endopeptidase activity	4/82	14/18410	3.54E-07	3.82E-05	3.05E-05	PSMB9/PSMB8/PSMB10/PSMA4	4
MF	GO:00700	threonine-type peptidase activity	4/82	24/18410	3.63E-06	0.00026132	0.000208853	PSMB9/PSMB8/PSMB10/PSMA4	4

ONTOLOGY	ID	Description	GeneRatio	BgRatio	pvalue	p.adjust	qvalue	geneID	Count
	03					1			
MF	GO:0003823	antigen binding	7/82	174/18410	1.25E-05	0.000675161	0.000539602	TAP1/TAP2/HLA-F/HLA-B/LAG3/HLA-C/HLA-A	7
MF	GO:0042379	chemokine receptor binding	5/82	71/18410	1.60E-05	0.000692219	0.000553236	STAT1/CXCL10/CCL5/CXCL9/CXCL11	5
MF	GO:1904680	peptide transmembrane transporter activity	3/82	15/18410	3.73E-05	0.001342226	0.001072734	TAP1/TAP2/SLC15A3	3

Table S4. The enrichment analysis of HALLMARK terms were analyzed by GSEA in pan-cancer.

ID	setSize	enrichmentScore	NES	pvalue	p.adjust	qvalue	rank	leading_edge	core_enrichment
HALLMARK_HEDGEHOG_SIGNALING	36	-0.713653042	-2.156557144	0.016393443	0.030637255	0.007094943	4155	tags=56%, list=12%, signal=49%	NKX6-1/RASA1/CDK5R1/NF1/VLDLR/UNC5C/L1CAM/PTCH1/ADGRG1/HEY1/OPHN1/HEY2/AMOT/RTN1/DPYSL2/CNTFR/SLIT1/CRMP1/SCG2/NRCAM
HALLMARK_MYOGENESIS	198	-0.416306465	-1.692485523	0.025641026	0.036630037	0.008482745	4111	tags=32%, list=11%, signal=28%	ACTC1/CD36/SPTAN1/COX7A1/IGFBP7/SIRT2/MEF2D/WWTR1/SGCD/NAV2/APP/APOD/FOXO4/ABLIM1/HDAC5/PPFIA4/SPARC/SPEG/MAPRE3/ADCY9/SCHIP1/MYLK/PRNP/HRC/CASQ2/MYH7/MYOM2/CKMT2/CDH13/MYH11/PFKM/CASQ1/LDB3/MYOZ1/MEF2A/ACTN2/MYOM1/MYL3/APLNR/DMD/SSPN/REEP1/CACNA1H/MRAS/KCNH2/CKB/FABP3/PYGM/MEF2C/FGF2/PPP1R3C/ITGA7/PKIA/CAMK2B/HSPB8/GNAO1/SORBS1/GPX3/DTNA/CLU/NCAM1/FHL1/CRYAB
HALLMARK_UV_RESPONSE_DN	144	-0.436307947	-1.689044469	0.020408163	0.030921459	0.007160759	4878	tags=40%, list=14%, signal=35%	PRKAR2B/RBPMS/PDLIM5/PHF3/KCNMA1/CACNA1A/TJP1/MGLL/ATXN1/DAB2/GJA1/NR1D2/RGS4/SCN8A/NOTCH2/WDR37/TOGARAM1/ATRX/APBB2/BCKDHB/TGFBR3/ATP2B4/DUSP1/AMPH/CDC42BPA/ITGB3/VLDLR/SCHIP1/ADD3/SDC2/CDON/PLCB4/KALRN/NR3C1/CELF2/CAP2/PTPN21/ADGRL2/PRKCE/IGFBP5/ATRN/CITED2/IGF1R/NFIB/ARHGEF9/DDAH1/MAGI2/DLC1/ZMIZ1/PRKCA/SYNE1/FYN/MMP16/PTPRM/KIT/PLPP3/AKT3/MAP1B
HALLMARK	39	-0.5361	-1.676	0.0161	0.0306	0.0070	55	tags=44%,	PCSK1/SLC2A2/NEUROD1/NKX6-1/LMO2/ABCC8/NKX2-

ID	setSize	enrichmentScore	NES	pvalue	p.adjust	qvalue	rank	leading_edge	core_enrichment
_PANCREAS_BETA_CELLS		43109	609594	29032	37255	94943	49	list=15%, signal=37%	2/INSM1/DCX/CHGA/PAK3/SCGN/STXBP1/SYT13/SST/AKT3/PCSK2
HALLMARK_HYPOXIA	197	0.354230012	1.40666641	0.047619048	0.061050061	0.014137909	4629	tags=32%, list=13%, signal=28%	ISG20/S100A4/PLAUR/PKP1/ANXA2/SULT2B1/HOXB9/TGFBI/IER3/COL5A1/PLAC8/TPBG/CASP6/LDHA/TNFAIP3/EDN2/ERO1A/ADORA2B/MT2A/KDELR3/SDC4/TES/DCN/EFNA1/FBP1/ENO1/SIAH2/EFNA3/EXT1/TGM2/GALK1/PIM1/TPI1/PRDX5/P4HA2/SLC2A1/F3/BHLHE40/ADM/SELENBP1/IL6/HSPA5/DDIT4/IGFBP3/HK2/FAM162A/TPD52/TMEM45A/PGK1/CAVIN3/BGN/GLRX/CXCR4/GPI/MT1E/SERPINE1/CCN5/GPC3/PGM2/STC2/GAPDH/CP/MYH9/JMJD6
HALLMARK_G2M_CHECKPOINT	200	0.765854581	3.05121858	0.015625	0.030637255	0.007094943	4057	tags=56%, list=11%, signal=50%	UBE2C/CDC20/MYBL2/BIRC5/AURKB/PTTG1/CDKN3/TPX2/CCNB2/NEK2/CDK1/KIF2C/CENPA/CCNA2/TOP2A/TROAP/CDC45/PBK/AURKA/PLK1/CKS2/MKI67/NDC80/KIF4A/LMNB1/CDC6/TACC3/HMMR/BUB1/EXO1/KIF23/TTK/KIF11/GINS2/CENPF/JPT1/KPNA2/CKS1B/BCL3/UBE2S/HMGA1/E2F2/MAD2L1/EZH2/ESPL1/CCNF/ORC6/E2F1/HMGB3/H2AZ1/CENPE/KIF15/RACGAP1/PLK4/CDC25A/STIL/H2AX/RAD54L/CHEK1/MCM6/KNL1/H2BC12/DTYMK/TRAIP/DDX39A/MCM5/CDC25B/POLQ/POLA2/CHAF1A/ATF5/STMN1/MCM3/TMPO/KIF22/SQLE/SUV39H1/DBF4/SNRPD1/MT2A/MCM2/CDC7/NASP/SLC7A5/FBXO5/HMGN2/SMC4/DKC1/KIF20B/BUB3/PRIM2/POLE/PML/SRSF2/BRCA

ID	setSize	enrichmentScore	NES	pvalue	p.adjust	qvalue	rank	leading_edge	core_enrichment
									2/CDKN2C/MNAT1/RBL1/MYC/LBR/TFDP1/INCENP/BARD1/ODF2/EFNA5/E2F4/NOLC1/HNRNPD/UCK2/SMC2/NCL
HALLMARK_INTERFERON_ALPHA_RESPONSE	97	0.86341961	3.095682412	0.018181818	0.030637255	0.007094943	3230	tags=76%, list=9%, signal=70%	CXCL10/IFI27/CXCL11/ISG15/BST2/OASL/IFITM1/PSMB9/PSME2/IRF1/OAS1/LAMP3/MX1/EPSTI1/BATF2/TAP1/PSMB8/RTP4/IFI35/ISG20/IFI44/IFITM3/LY6E/IRF7/GBP4/PSME1/C1S/RSAD2/NMI/WARS1/CASP1/HLA-C/UBE2L6/CD74/IFIT3/PARP12/LGALS3BP/GBP2/IFIH1/IFI44L/CMPK2/USP18/B2M/PARP9/TRIM21/DDX60/SAMD9/PLSCR1/PARP14/HERC6/LAP3/PSMA3/SP110/MOV10/HELZ2/UBA7/SAMD9L/DHX58/IL4R/IFITM2/TRIM14/CASP8/IFIT2/IRF9/IL15/CCRL2/OGFR/RIPK2/IL7/TRAFD1/STAT2/PROCR/SLC25A28/TRIM26
HALLMARK_ALLOGRAFT_REJECTION	199	0.780161613	3.101638672	0.015873016	0.030637255	0.007094943	3510	tags=62%, list=10%, signal=56%	CXCL9/CXCL13/GZMB/CD3D/CCL5/GZMA/CD2/MMP9/CCL19/CD3E/LTB/IL2RG/CD7/CD8A/LCK/CXCR3/TAP1/SIT1/CD79A/PRF1/PSMB10/IRF7/CCL13/C2/STAT1/IL2RB/WARS1/HLA-DQA1/HLA-A/CD247/SOCS1/HLA-DRA/CD74/BCL3/CCL22/TAP2/GBP2/ZAP70/IFNG/CD8B/CCR5/HLA-DMA/FASLG/CD3G/CDKN2A/CAPG/B2M/CTSS/CCL11/IL2RA/HLA-DOB/CCL4/TIMP1/IL12RB1/CD96/ITGAL/CD40/NME1/PTPN6/HLA-G/NCF4/HLA-DMB/TAPBP/LY75/HLA-E/CRTAM/RPS19/TRAF2/ICAM1/ELF4/IL18/CCL7/TRAT1/IL27RA/IL4R/IRF8/ITK/MAP4K1/EIF5A/HLA-DOA/ITGB2/CD86/PTPRC/WAS/TNF/IRF4/CD80/HCLS1/CD4/SPI1/CCL2/I

ID	setSize	enrichmentScore	NES	pvalue	p.adjust	qvalue	rank	leading_edge	core_enrichment
									NHBA/IL11/EREG/IL15/IGSF6/BRCA1/CD28/BCL10/LCP2/CSK/RARS1/FCGR2B/CCR1/GCNT1/RIPK2/IL7/IFNGR2/RPS9/CD40LG/FYB1/IL1B/IL18RAP/IL6/NPM1/KRT1/LIF/GPR65/CCR2/LYN/STAT4/FAS/TPD52
HALLMARK_E2F_TARGETS	200	0.818253386	3.259979106	0.015625	0.030637255	0.007094943	4175	tags=72%, list=12%, signal=64%	CDC20/MYBL2/BIRC5/TK1/AURKB/RRM2/DLGAP5/PTTG1/CDKN3/ASF1B/CCNB2/MELK/CDK1/KIF2C/CDCA8/CENPM/TOP2A/AURKA/PLK1/CKS2/UBE2T/MKI67/SPC24/KIF4A/LMNB1/TACC3/HMMR/CDCA3/TRIP13/SPC25/JPT1/KPNA2/KIF18B/CKS1B/UBE2S/HMGA1/DEPDC1/GINS1/SPAG5/BUB1B/MAD2L1/E2F8/EZH2/ESPL1/CCNE1/ORC6/CDKN2A/HMGB3/PCNA/H2AZ1/RAD51AP1/HMGB2/SNRPB/CENPE/TCF19/RACGAP1/PLK4/CHEK2/CDC25A/MXD3/H2AX/CHEK1/MCM6/MCM4/TIMELESS/DSCC1/NME1/DDX39A/MCM5/CDC25B/TFRC/POLA2/MCM7/ATAD2/DIAPH3/POLE4/HELLS/STMN1/MCM3/TMPO/LYAR/KIF22/SUV39H1/DCTPP1/POLD1/RPA3/LIG1/RANBP1/TUBG1/MCM2/RFC2/NASP/TUBB/PRDX4/POP7/GINS4/PA2G4/RAN/RFC3/SMC4/PHF5A/NCAPD2/CTPS1/MTHFD2/DONSON/BRCA1/GINS3/PRIM2/POLE/SLBP/CSE1L/DNMT1/SRSF2/BRCA2/DUT/NOP56/RBBP7/NUP107/TBRG4/POLD2/CDKN2C/EIF2S1/DEK/EED/TIPIN/UNG/AK2/PAICS/XRCC6/CNOT9/MYC/LBR/EXOSC8/BARD1/POLD3/DCLRE1B/NOLC1/HNRNPD/NAA38/USP1/WEE1/TP53/SSRP1

ID	setSize	enrichmentScore	NES	pvalue	p.adjust	qvalue	rank	leading_edge	core_enrichment
HALLMARK_INTERFERON_GAMMA_RESPONSE	200	0.832257054	3.315770707	0.015625	0.030637255	0.007094943	3484	tags=70%, list=10%, signal=64%	CXCL10/IFI27/CXCL9/CXCL11/IDO1/ISG15/BST2/CCL5/OASL/GZMA/PSMB9/SECTM1/PSME2/OAS2/IRF1/MX1/EPSTI1/BATF2/TAP1/SLAMF7/PSMB8/HLA-B/RTP4/OAS3/IFI35/ISG20/TNFSF10/IFI44/PSMB10/IFITM3/LY6E/IRF7/HLA-DRB1/C1R/STAT1/IL2RB/GBP4/PSME1/C1S/IL15RA/RSAD2/NMI/WARS1/CASP1/HLA-DQA1/HLA-A/TNFAIP2/SOCS1/UBE2L6/MX2/CD74/IFIT3/PARP12/GCH1/LGALS3BP/ZBP1/VAMP8/NLRC5/IFIH1/IFI44L/HLA-DMA/CMPK2/USP18/B2M/CASP4/APOL6/TRIM21/DDX60/CD274/DDX58/CASP7/PLSCR1/XCL1/PARP14/HERC6/IFIT1/LAP3/CD40/PSMA3/PTPN6/HLA-G/SP110/GBP6/HELZ2/XAF1/TAPBP/MVP/TNFAIP3/SAMD9L/DHX58/ICAM1/CCL7/IL4R/IRF8/SAMHD1/SERPING1/MT2A/IFITM2/TRIM14/CD86/MYD88/CIITA/CASP8/SOCS3/CD69/CFB/IRF4/VAMP5/PSMB2/CFH/CCL2/PNP/MTHFD2/IFIT2/IRF9/CASP3/IL15/IL18BP/NFKBIA/PML/RNF213/LCP2/PIM1/OGFR/CSF2RB/GPR18/RIPK2/IL7/IL10RA/PTPN2/RBCK1/TRAFD1/STAT2/IL6/UPP1/SLC25A28/TRIM26/STAT4/ITGB7/FCGR1A/FAS

Table S5. Genes involved in oncogenic pathways.

Chromatin modifiers status	HIPPO pathway status	RTK pathway status	SWI/SNF complex status
CREBBP	NF2	BRAF	ACTB
EHMT1	SAV1	EGFR	ACTL6A
EHMT2	WWC1	ERBB2	ACTL6B
EP300		ERBB3	ARID1A
EZH1		ERBB4	ARID1B
EZH2		FGFR1	ARID2
KAT2A		FGFR2	BCL11A
KAT2B		FGFR3	BCL11B
KDM1A		FGFR4	BCL6
KDM1B		HRAS	BCL6B
KDM4A		KIT	BRD7
KDM4B		KRAS	BRD9
KDM5A		MET	DPF1
KDM5B		NF1	DPF2
KDM5C		NRAS	DPF3
KDM6A			PBRM1
KDM6B			PHF10
KMT2A			SMARCA2
KMT2B			SMARCA4
KMT2C			SMARCB1
KMT2D			SMARCC1
KMT2E			SMARCC2
NSD1			SMARCD1
SETD2			SMARCD2
SMYD4			SMARCD3
SRCAP			SMARCE1

Table S6. The association between PSME2 promoter methylation and M1 macrophage infiltration.

Cancer Type	Macrophage (ssGSEA)		Macrophage (xCell)		Macrophage M1 (CIBERSORT)		Macrophage M1 (xCell)		Macrophage M2 (xcell)	
	P-value	Cor	P-value	Cor	P-value	Cor	P-value	Cor	P-value	Cor
ACC	0.024	0.259	0.01	0.295	0.674	-0.067	0.007	0.306	0.002	0.345
BLCA	0	-0.183	0	-0.204	0.168	-0.073	0	-0.207	0.018	-0.118
BRCA	0.138	-0.046	0.011	-0.078	0.844	-0.006	0.003	-0.091	0.383	-0.027
CESC	0.946	-0.004	0.671	-0.025	0.132	-0.088	0.474	-0.042	0.255	-0.067
CHOL	0.758	0.054	0.717	-0.063	0.286	-0.191	0.913	-0.019	0.256	-0.197
COAD	0.398	-0.052	0.092	-0.102	0.361	-0.056	0.048	-0.119	0.747	-0.02
DLBC	0.923	-0.014	0.883	0.022	0.581	-0.082	0.674	0.063	0.831	-0.032
ESCA	0.044	-0.161	0.154	-0.115	0.036	-0.17	0.085	-0.138	0.462	-0.059
GBM	0.292	-0.095	0.152	-0.13	0.292	0.097	0.781	-0.025	0.463	-0.067
HNSC	0.002	0.141	0.019	0.105	0.148	0.065	0.034	0.095	0.082	0.078
KICH	0.158	0.179	0.165	0.176	0.39	-0.12	0.581	0.07	0.465	0.093
KIRC	0.588	-0.024	0.022	0.102	0.084	-0.077	0.335	0.043	0.034	0.094
KIRP	0.019	-0.14	0.09	-0.102	0.658	0.028	0.408	-0.05	0.017	-0.143
LAML	NA	NA	0.428	0.072	0.327	0.088	0.068	0.164	0.917	-0.009
LGG	0.023	0.101	0.028	0.097	0.011	0.133	0.041	0.091	0.765	0.013
LIHC	0	0.241	0.001	0.181	0.24	-0.064	0.008	0.139	0.497	0.036
LUAD	0.643	0.021	0.746	-0.015	0.637	-0.021	0.789	0.012	0.999	0
LUSC	0.953	-0.003	0.53	-0.029	0.645	0.021	0.495	-0.032	0.456	-0.035
MESO	0.046	-0.216	0.106	-0.175	0.114	-0.172	0.028	-0.237	0.173	-0.148
OV	0.014	-0.153	0.205	-0.079	0.033	0.138	0.245	-0.072	0.183	-0.083
PAAD	0.625	-0.037	0.241	-0.089	0.845	0.015	0.044	-0.151	0.121	-0.117
PCPG	0.622	-0.037	0.602	-0.04	0.263	-0.111	0.55	-0.045	0.106	-0.122
PRAD	0.658	-0.02	0.876	0.007	0.002	0.15	0.549	-0.027	0.333	-0.044

Cancer Type	Macrophage (ssGSEA)		Macrophage (xCell)		Macrophage M1 (CIBERSORT)		Macrophage M1 (xCell)		Macrophage M2 (xcell)	
	P-value	Cor	P-value	Cor	P-value	Cor	P-value	Cor	P-value	Cor
READ	0.061	-0.204	0.028	-0.238	0.492	0.076	0.01	-0.277	0.272	-0.121
SARC	0.838	-0.013	0.992	0.001	0.917	-0.007	0.737	-0.021	0.524	0.04
SKCM	0.952	0.003	0.885	0.007	0.012	0.125	0.959	0.002	0.489	-0.034
STAD	0	0.26	0.018	0.124	0.483	0.037	0.069	0.096	0.005	0.148
TGCT	0.043	0.177	0.322	0.087	0.018	-0.208	0.014	0.213	0.504	0.059
THCA	0.039	-0.093	0.959	0.002	0.871	0.008	0.667	0.019	0.297	0.047
THYM	0.686	-0.038	0.875	-0.015	0.007	-0.251	0.494	0.064	0.772	-0.027
UCEC	0.693	0.03	0.49	0.053	0.083	0.137	0.864	-0.013	0.255	0.087
UCS	0.183	0.179	0.782	0.037	0.903	0.018	0.971	-0.005	0.888	-0.019
UVM	0.968	0.005	0.236	-0.135	0.27	-0.174	0.271	-0.125	0.84	-0.023

Table S7. The correlation between PSME2 expression and 14 cancer functional statesfrom the CancerSEA database.

Cancer	Angiogenesis	Apoptosis	CellCycle	Differentiation	DNAdamage	DNArepair	EMT	Hypoxia	Inflammation	Invasion	Metastasis	Proliferation	Quiescence	Stemness
ALL	-0.25	-0.078	0.1	-0.083	0.072	0.197	-0.06	-0.129	-0.067	-0.012	0.043	-0.026	-0.024	-0.011
AML	0.091	-0.021	0.093	0.082	0.046	0.094	0.12	-0.043	0.071	0.208	0.136	0.081	-0.035	0.016
CML	-0.059	0.032	0.189	0.014	0.096	0.24	0.102	0.065	-0.162	0.262	0.106	0.197	-0.151	-0.053
CRC	0.049	-0.021	0.059	0.003	-0.068	-0.048	0.018	-0.062	0	0.038	0.045	0.058	-0.085	0.013
BRCA	-0.268	-0.018	0.263	-0.186	0.272	0.405	-0.009	-0.133	-0.064	0.228	-0.035	0.12	-0.176	-0.186
AST	0.024	0.006	0.077	0.009	0.058	0.004	0.08	0.043	0.007	0.057	0.091	0.007	0.011	-0.023
GBM	-0.075	0.061	0.088	-0.064	0.126	0.125	0.106	0.046	-0.082	0.207	0.078	-0.02	0.044	0.068
Glioma	-0.022	0.108	0.198	0.102	0.082	0.089	0.005	0.104	0.152	0.172	0.222	0.024	0.148	-0.006
HGG	-0.14	-0.008	0.263	0.038	0.166	0.081	-0.079	-0.132	-0.107	0.005	-0.012	0.139	-0.153	0.178
ODG	-0.024	-0.033	0.077	0.055	-0.073	0.024	0.052	0.101	0.097	0.006	0.077	0.017	0.046	0.05
HNSCC	0.07	0.063	0.278	0.095	0.208	0.215	0.17	0.04	-0.041	0.389	0.164	0.357	0.036	-0.155
RCC	-0.152	-0.059	0.288	-0.092	-0.082	0.244	-0.074	-0.299	0.146	-0.072	-0.096	0.078	0.083	-0.152
LUAD	-0.004	0.044	0.206	-0.016	0.28	0.246	0.14	-0.134	-0.177	0.274	0.036	0.144	-0.094	0.099
NSCLC	-0.323	-0.025	0.42	-0.094	0.351	0.346	-0.163	-0.354	-0.297	0.347	-0.357	0.285	-0.211	0.095
OV	0.047	-0.002	0.039	0.031	0.071	-0.069	-0.148	-0.024	0.067	-0.067	-0.147	0.036	0.06	-0.028
PC	0.19	0.137	-0.11	-0.181	-0.216	0.118	-0.312	-0.058	-0.17	0.333	-0.246	-0.082	-0.113	-0.351
MEL	-0.111	0.018	0.204	0.058	0.12	0.13	-0.049	-0.122	-0.068	0.075	0.029	0.214	-0.003	0.092
RB	0.315	-0.105	-0.263	0.354	-0.251	-0.286	-0.037	-0.092	0.216	0.008	0.113	-0.058	0.112	0.132
UM	-0.14	-0.445	-0.277	-0.305	-0.464	-0.491	-0.249	-0.392	-0.284	-0.405	-0.43	-0.217	-0.416	-0.162

Table S8. Synergistic effect between irinotecan and paclitaxel.

Symbol	Irinotecan(μM)	Paclitaxel(μM)	Combination index (CI)
1	0.01	0.01	0.373
2	0.1	0.1	0.308
3	1	1	0.688
4	0.01	0.01	0.512
5	0.1	0.1	0.482
6	1	1	0.347
